# Electronic Health Record Driven Prediction for Gestational Diabetes Mellitus in Early Pregnancy

**DOI:** 10.1038/s41598-017-16665-y

**Published:** 2017-11-27

**Authors:** Hang Qiu, Hai-Yan Yu, Li-Ya Wang, Qiang Yao, Si-Nan Wu, Can Yin, Bo Fu, Xiao-Juan Zhu, Yan-Long Zhang, Yong Xing, Jun Deng, Hao Yang, Shun-Dong Lei

**Affiliations:** 1 Big Data Research Center, University of Electronic Science and Technology of China, Chengdu, 611731 Sichuan China; 2 School of Computer Science and Engineering, University of Electronic Science and Technology of China, Chengdu, 611731 Sichuan China; 3 School of Economics and Management, Chongqing University of Posts and Telecommunications, Chongqing, 400065 Chongqing, China; 4 Department of Statistics, The Pennsylvania State University, University Park, PA 16802-2111 United States; 5 Division of Obstetrics, West China Second University Hospital, Sichuan University, Chengdu, 610041 Sichuan China; 6 Division of Information Management, West China Second University Hospital, Sichuan University, Chengdu, 610041 Sichuan China; 7 Chengdu Shulianyikang Technology Co., Ltd, Chengdu, 610041 Sichuan China; 8 School of Computer Science, Chengdu University of Information Technology, Chengdu, 610225 Sichuan China

## Abstract

Gestational diabetes mellitus (GDM) is conventionally confirmed with oral glucose tolerance test (OGTT) in 24 to 28 weeks of gestation, but it is still uncertain whether it can be predicted with secondary use of electronic health records (EHRs) in early pregnancy. To this purpose, the cost-sensitive hybrid model (CSHM) and five conventional machine learning methods are used to construct the predictive models, capturing the future risks of GDM in the temporally aggregated EHRs. The experimental data sources from a nested case-control study cohort, containing 33,935 gestational women in West China Second Hospital. After data cleaning, 4,378 cases and 50 attributes are stored and collected for the data set. Through selecting the most feasible method, the cost parameter of CSHM is adapted to deal with imbalance of the dataset. In the experiment, 3940 samples are used for training and the rest 438 samples for testing. Although the accuracy of positive samples is barely acceptable (62.16%), the results suggest that the vast majority (98.4%) of those predicted positive instances are real positives. To our knowledge, this is the first study to apply machine learning models with EHRs to predict GDM, which will facilitate personalized medicine in maternal health management in the future.

## Introduction

In developing regions, antenatal care increased from 65% in 1990 to 83% in 2012^1^. Although more women are receiving antenatal care, gestational diabetes mellitus (GDM) defined as glucose intolerance first recognized during pregnancy^2^, is still one of the most common medical complications of pregnancy^3^. According to the report of International Diabetes Federation (IDF)^4^, the total prevalence of GDM reaches almost 1% to 14% worldwide in 2014. In China, the recorded prevalence of GDM has increased from about 5% to more than 16% since the implementation of a new method of diagnosing GDM in December 2011^5^. Moreover, GDM increases the risk of development of type 2 diabetes mellitus in both mother and child^6^, is also associated with adverse short-term fetal outcomes and offspring long-term greater adiposity.

According to the International Association of the Diabetes and Pregnancy Study Groups (IADPSG) guidelines^7,8^, the screening and diagnosis of GDM can routinely be executed at the period of 24–28 weeks’ gestation. The pregnant women underwent routine second-trimester screening, namely oral glucose tolerance test (OGTT), for GDM according to the risk factor screening guideline^7^. A technique with a high sensitivity to predicate GDM at the first-trimester^9^ would be well-received for the clinical practioners and almost all pregnant women, decreasing the future risks of development of GDM.

Many studies of predicting modelling techniques^10^ have been conducted in the context of prospective cohort studies in which patients are followed up routinely by the investigators^11^. Wei, B. *et al*.^12^ studied parental smoking during pregnancy and presented log-binomial models with generalized estimating equations to predict relative risks of GDM in the daughter. However, those studies on risk factor analysis did not consider the details of gestational trimesters. To further investigate first-trimester prediction of GDM, Leng, J. *et al*.^13^ studied plasma levels of alanine aminotransferase, identifying high risk women for gestational diabetes. Savvidou, M. *et al*.^14^ presented a model by examining the potential of combining maternal characteristics and laboratory measures in the first trimester. In their study, the samples and dimensions of the data set were limited. They only investigated 124 maternal samples who developed GDM and 248 control subjects and they just measured no more than 20 parameters, including lipids, high-sensitivity C-reactive protein, adiponectin, etc. Despite of those recent progress, few studies have focused on the prediction for GDM with high dimensional electronic medical records (EHRs)^15,16^.

In China, prenatal examination provides regular check-ups that allow clinical physicians and midwives to treat and prevent potential health problems throughout the course of the pregnancy while promoting healthy lifestyles that benefit both mothers and children. Through prenatal examination, a mount of maternal characteristics data are collected and stored in EHRs, while these examination items vary from different pregnancy stages as well as their frequencies. Some clinical characteristics during pregnancy have been identified as principal risk factors for GDM, such as age, BMI, etc. Accumulating evidence in literature^17,18^ also indicated that those characteristics play important roles in predicting the development and progression of GDM^19^. Secondary analysis of those data broadens the way to predict incidence risk of GDM by digital health technology, such as machine learning. With high dimensional data from EHRs, Bertsimas D *et al*.^20^ investigated k-nearest neighbour algorithm for personalized diabetes management that improved health outcomes relative to the standard of care. They prescribed the regimen with best predicted outcomes from switching regimens and simulated the potential effects of recommendations on matched patient outcomes, while those models could not be directly used to predict GDM risks of gestational women.

Secondary analysis of EHRs promises to advance clinical research and better inform clinical decision making, but challenges in temporal representation and system’s discrimination ability of EHRs prevent widespread practice of predictive modelling^21^. Although there exist standard statistical methods for attribute reduction in prospective cohort studies, they cannot be directly applied to EHRs data, especially for analysing the progression of GDM. Instead of black box modelling, it would be interesting for the medical community to know the significant features. Meanwhile, it is meaningful to use all the attributes of the input data. The target disease (GDM) is often occurrence with many complications (i.e., excessive birth weight, hypoglycemia), which may be caused by the other attributes removed from the single task data set of GDM prediction. Although it is true for prospective cohort studies, in most other clinical scenarios and in EHRs, gestational women typically visit hospitals irregularly. Highly dimensional missing values and class-imbalanced data are prevailing phenomena in the irregularly spaced data of EHRs. Many statistical methods require balanced panel data and/or equidistant time series to analyze temporal phenomena. To improve the performance of the prediction model on imbalanced data^22^, cost-sensitive learning was taken as a potential method. Moreover, since physiological parameters of pregnant women vary from different stages of pregnancy, the values of data-driven predictions fail for a long period data set. To our knowledge, the development and application of machine learning algorithms (especially ensemble methods^23^) to predict GDM have not been reported. We therefore conducted this research with EHRs to identify the most feasible algorithm for predicting GDM, potentially advancing the diagnosis period of GDM and prognosis of its outcomes.

## Materials and Methods

**Figure 1:**
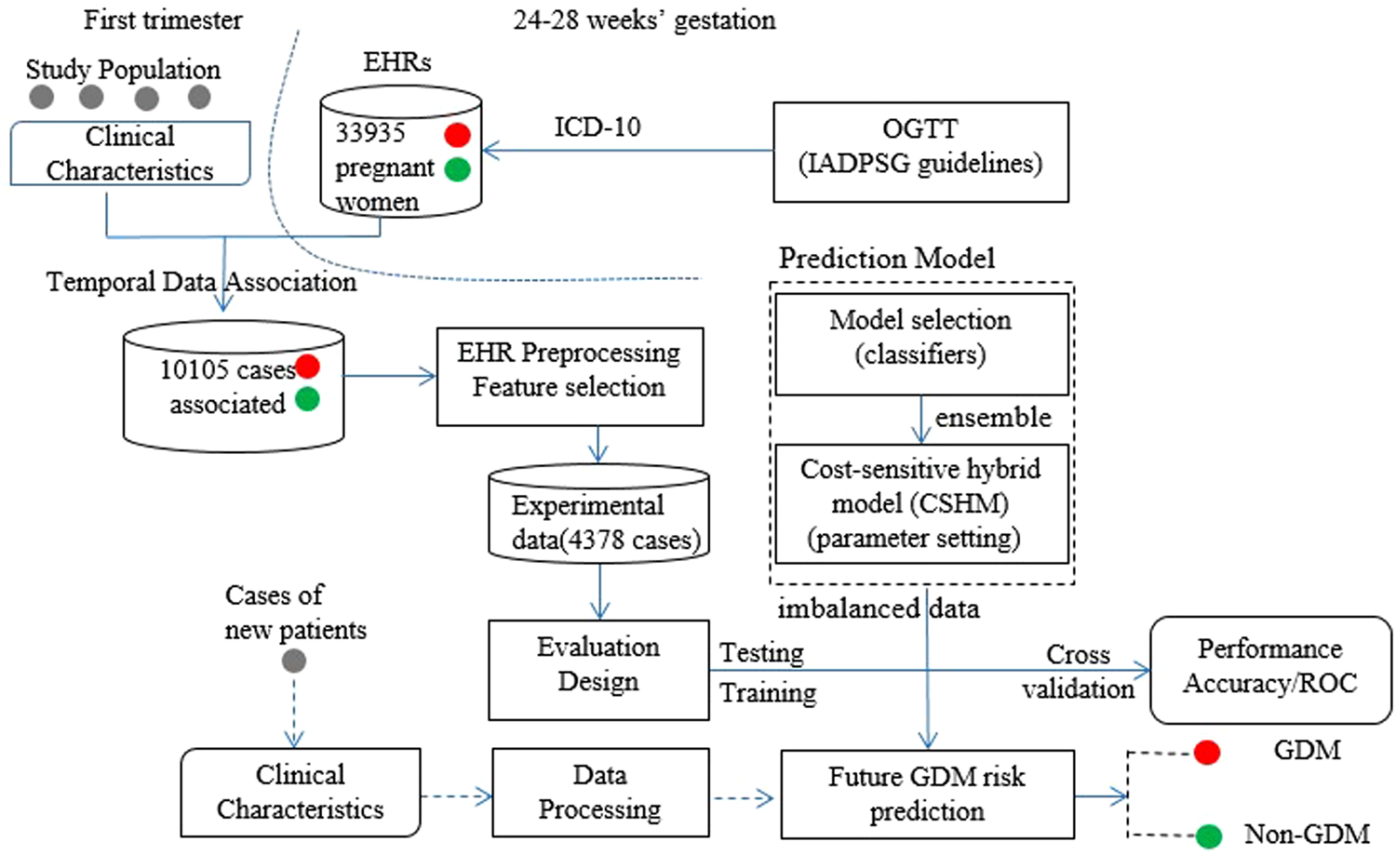
Prediction model and data processing schematic diagram. In EHRs, the feature vectors were extracted from the characteristics of the first trimester and the class labels from the diagnostic international classification of diseases (ICD-10) codes of OGTT in 24–28 weeks’ gestation. After EHR preprocessing, the experimental data were divided into two subsets in evaluation design. The training set was then modelled using six machine learning techniques and the variants of cost-sensitive hybrid models (CSHM). Five measure metrics of the performance were collected: accuracy; area under the ROC curve (AUC), true positive rates, false positive rates and confidence reports.

Our prediction framework was based on supervised learning^23^ (e.g., classification). Figure 1 shows a schematic representation of the prediction framework and its data processing steam in capturing temporal correlation and regularities in the aggregated EHRs. We implemented two steps to complete the task of prediction model construction. First, in model selection step, five conventional machine learning classifiers and a variant of ensemble learning model were used and compared to identify the most feasible model for predicting future risks of GDM. Then, in parameter setting step, cost-sensitive hybrid model was employed to deal with imbalance of the data, aiming to improve its effectiveness in classification.

### Basic Characteristics of the Study population

The EHRs data of our investigations were stored in our centred repository, which has been collected and managed by the West China Second Hospital in Chengdu, Sichuan. The experimental data was a nested case-control study cohort. In total, 33,935 gestational women were enrolled in the EHRs from year 2013 to 2016. GDM related information of those samples contained 106 features of archiving data, 23 features of inspection data, 157 features of test data from laboratory information system (LIS) and 268 features of the first pages of EHRs. After data cleaning, we used a filtering strategy to preselect patients as our candidate samples whose EHRs data were related to GDM, excluding those of pregestational diabetes mellitus (PGDM). Through this process, we obtained a concrete data set of 10,105 samples with common clinical characteristics. In this data base, there were 1,649 GDM (positive) cases and 8,456 Non-GDM (negative) cases. However, this sample dataset still existed massive missing values, due to the various inspection terms among different patients. To make the dataset fit for classification, we removed the samples and attributes with their missing values over a certain level (i.e., 50%). Then, 4,378 cases with 50 attributes (less than 10% of the total attributes) remained for further EHRs data processing and machine learning experiments, as shown in Table 1.

**Table 1:** Statistical description of the sample attributes.

index	field	Description	#. values	#. Missing	mean	media	Mode	s.d.	variance	Minimum	Maximum
1	high_risk	High Risk Pregnancy (Age Over 35)	4378	0	0.13	0.00	0	0.336	0.113	0	1
2	marriage_ages	Marriage Years	4213	165	3.93	3.00	1	3.687	13.596	0	26
3	height	Height	4374	4	160.14	160.00	160	4.799	23.034	130	177
4	pregnancy_times	Pregnancy Times	4378	0	2.13	2.00	1	1.330	1.770	0	12
5	husband_age	Husband Age	4372	6	32.19	31.00	29	4.898	23.994	21	64
6	delivery_age	Production Age	4374	4	30.53	30.00	28	3.926	15.415	19	47
7	bmi	Body Mass Index (BMI)	4373	5	20.9005	20.5700	20.70	2.59699	6.744	14.62	36.89
8	Nonnative	Nonnative	4298	80	0.12	0.00	0	0.325	0.106	0	1
9	HCT	Hematocrit	4378	0	1.79	2.00	2	0.409	0.167	1	3
10	MCH	The Level Of Mean Corpsular Hemoglobin	4378	0	2.28	2.00	2	0.557	0.310	1	3
11	WBC	Count of White Blood Cell	4378	0	2.17	2.00	2	0.380	0.144	1	3
12	EOS	Eosinophils	4378	0	1.55	2.00	2	0.508	0.258	1	3
13	MPV	Mean Platelet Volum	4378	0	2.10	2.00	2	0.297	0.088	2	3
14	PDW	Platelet Distribution Width	4378	0	2.07	2.00	2	0.248	0.061	2	3
15	RDW.CV	Red Blood Cell Distribution Width CV	4378	0	1.84	2.00	2	0.545	0.297	0	2
16	RDW.SD	Red Blood Cell Distribution Width SD	4378	0	2.18	2.00	2	0.385	0.148	1	3
17	MONO.	Monocytes	4378	0	1.94	2.00	2	0.255	0.065	1	3
18	EOS.	Eosinophil	4341	37	1.67	2.00	2	0.487	0.237	1	3
19	PCT	Path CAST	4378	0	2.05	2.00	2	0.227	0.052	1	3
20	P.LCR	Platelet-Large Cell Rate	4378	0	2.10	2.00	2	0.293	0.086	2	3
21	HBsAg	Hepatitis B[Virus] Surface Antigen	4378	0	1.88	2.00	2	0.475	0.226	0	2
22	Anti.HBs	Hepatitis B Surface Antibody	4378	0	.51	.00	0	0.874	0.764	0	2
23	Anti.HBe	Hepatitis B E Antibody	4378	0	1.69	2.00	2	0.727	0.529	0	2
24	HBcAb.T.	Hepatitis B Core Antibody	4378	0	1.55	2.00	2	0.838	0.702	0	2
25	ALT	Alanine Aminotransferase	4378	0	1.83	2.00	2	0.557	0.310	0	2
26	AST	Aspartate Transaminase	4378	0	1.88	2.00	2	0.470	0.221	0	2
27	PA	Prealbumin	4375	3	1.75	2.00	2	0.436	0.190	1	2
28	UN	Urea	4378	0	1.43	1.00	1	0.495	0.245	1	2
29	UA	Uric Acid	4378	0	1.82	2.00	2	0.384	0.147	1	3
30	FPG	Fasting Plasma Glucose	4370	8	1.90	2.00	2	0.294	0.087	1	3
31	RBC	Red Blood Cell	4378	0	2.30	2.00	2	0.458	0.209	2	3
32	EC	Epithelial Cell	4378	0	2.41	2.00	2	0.492	0.242	2	3
33	XYSPXB	Number of Small round epithelial cel	4378	0	2.90	3.00	3	0.294	0.087	2	3
34	CAST	Cast	4378	0	2.25	2.00	2	0.435	0.189	2	3
35	CAST.1	Pathological cast	4378	0	2.19	2.00	2	0.392	0.154	2	3
36	EC.1	Education	4378	0	2.40	2.00	2	0.491	0.241	2	3
37	WBC.1	White Blood Cell	4377	1	2.41	2.00	2	0.492	0.242	2	3
38	TPOAb	Antithyroid Peroxidase Autoantibody	4267	111	1.65	2.00	2	0.759	0.575	0	2
39	TSH3UL	Thyroid Stimulating Hormone - Hypersensitivity	4272	106	1.85	2.00	2	0.406	0.165	1	3
40	Anti.A	Anti-A Blood Grouping Reagents	4378	0	2.39	2.00	2	0.489	0.239	2	3
41	Anti.B	Anti-B Blood Grouping Reagents	4378	0	2.35	2.00	2	0.476	0.226	2	3
42	A1cells	A1cells	4378	0	2.61	3.00	3	0.489	0.239	2	3
43	Bcells	Bursa Oriented Cells	4378	0	2.65	3.00	3	0.476	0.227	2	3
44	RBC.1	Red Blood Cell Count	4378	0	1.91	2.00	2	0.345	0.119	1	3
45	LYMPH.	Lymphocyte	4378	0	1.31	1.00	1	0.463	0.214	1	3
46	NEUT	Neutrophil	4378	0	2.20	2.00	2	0.404	0.163	1	3
47	NEUT.	Neutrophilic Granulocyte	4378	0	2.89	3.00	3	0.312	0.097	2	3
48	r.GT	Glutamyl Transpeptidase	4378	0	1.87	2.00	2	0.487	0.237	0	2
49	ALP	Alkaline Phosphatase	4378	0	1.69	2.00	2	0.467	0.218	1	3
50	label_gdm	Gestational diabetes mellitus	4378	0	0.14	0.00	0	0.346	0.120	0	1

Note: #. values (missing) means the number of values (missing). s.d.: standard deviation. In most clinical scenarios, patients typically visit hospitals irregularly. Gestational women normally do not take all the tests and examinations when they visit hospitals. Oftentimes we only observe some phenotype information from a patient in each of her visit, resulting in missing values for the others. Thus missing values are a prevailing phenomenon in EHR data. In addition, EHR data are inherently highly dimensional and spread across multiple aspects of health care. Features have been carefully selected or constructed before the data analysis in order to achieve the best predictive performance. In order to ensure the stability of the predictive models, some features were removed prior to data imputation. Features presented in less than 50% of patients in an EHR cohort were discarded from our analysis. The attribute “Family History of Type 2 Diabetes” should also be considered for training the model. However, in the present information system of this hospital, it did not collect the data of this attribute.

### Instance Representation with Temporal Data Association

The acquisition period of the instances herein is the first time of registration of the gestational women in the hospital (no later than 13 weeks’ gestation), which is much earlier than that of identifying the class labels by OGTT (24–28 weeks’ gestation). We denote *t*_I_ and *t*_II_ as two periods of data association. The observations of instance *x*_*i*_(*t*_I_) are acquired at *t*_I_ and the associated labels of each instance, *c*_*i*_(*t*_II_), is identified at *t*_II_. Given a training data set

1 $$D({t}_{{\rm{I}}},\,{t}_{{\rm{II}}})={\{({x}_{i}({t}_{{\rm{I}}}),\,{c}_{i}({t}_{{\rm{II}}}))\}}_{i=1}^{N}$$

where *N* is the number of the instances and *c*_*i*_ (*t*_II_) ∈ {−1, +1}. We assume that *c*_*i*_ (*t*_II_) is a predicted value of *c*_*i*_ (*t*_I_).

The goal of learning is to construct a strategy or algorithm *π*, which satisfies the decision makers’ criterion. For example, maximizes the generalization accuracy,

2 $$Acc(\pi )={{\rm{E}}}_{x\sim D}[{\rm{\Pi }}(\pi (x)=f(x))]$$

where Π(·) is an indicator function and E(·) is its expectation when *x* obeys the distribution *D* and *f* the ground-truth target function.

Given characteristics *x*_*i*_ (*t*_I_) and label *c*_*i*_ (*t*_II_) in a sequence data set *D*(*t*_I_, *t*_II_), we train *π* in the form of “*x*_*i*_ (*t*_I_) → *c*_*i*_ (*t*_II_)”, meaning that characteristics *x*_*i*_ (*t*_I_) in the sequence implies label *c*_*i*_ (*t*_II_) is also in the sequence. And its confidence of *π* is defined as

3 $$Confidence(\pi )=\frac{\delta ({x}_{i}({t}_{{\rm{I}}})\,{\rm{a}}{\rm{n}}{\rm{d}}\,{c}_{i}({t}_{{\rm{I}}{\rm{I}}}))\,\,}{\delta ({x}_{i}({t}_{{\rm{I}}}))}$$

where *δ*(·) is the number of the characteristics. The confidence implies the proportion of training sequences with characteristics *x*_*i*_ (*t*_I_) that also have label *c*_*i*_ (*t*_II_).

Given a query instance data set *Q*, *q*_*s*_ (*t*_I_) ∈ *Q* acquired at *t*_I_ is regarded as a test instance, then its consequence label is deduced as *π* (*q*_*s*_ (*t*_I_)). This outcome is a predictive label for π(*q*_*s*_ (*t*_I_)) at *t*_II_. Since *c*_*s*_ (*t*_II_) is a predicted value of *c*_*s*_ (*t*_I_), π(*q*_*s*_ (*t*_II_)) = *c*_*s*_ (*t*_II_), if Π(*π* (*q*_*s*_ (*t*_I_)) = *f* (*q*_*s*_ (*t*_II_))) = 1, then Π(*π* (*q*_*s*_ (*t*_I_)) = *c*_*s*_ (*t*_II_)) = 1, or else Π(*π* (*q*_*s*_ (*t*_I_)) = *c*_*s*_ (*t*_II_)) = 0.

### Cost-sensitive Hybrid Model for Classifying

Since clinical data are often imbalanced and cost-sensitive, conventional methods can predict all the instances as negative with still high accuracy. However, this is not an ideal choice for those instances and in certain cirtuances, the cost of the positive instances are more sensitive than the negative. We assume that the minority class (positive) has higher cost than the majority class.

We suppose the cost of misclassifying the *i*th class to the *j*th class is *M*_*ij*_,

4 $${M}_{ij}=\{\begin{array}{cc}0 & \pi ({q}_{s}({t}_{{\rm{I}}}))={c}_{s}({t}_{{\rm{I}}{\rm{I}}})\\ {\lambda }_{1} & \pi ({q}_{s}({t}_{{\rm{I}}}))\ne {c}_{s}({t}_{{\rm{I}}{\rm{I}}}),\,{c}_{s}({t}_{{\rm{I}}{\rm{I}}})=-1\\ {\lambda }_{2} & \pi ({q}_{s}({t}_{{\rm{I}}}))\ne {c}_{s}({t}_{{\rm{I}}{\rm{I}}}),\,{c}_{s}({t}_{{\rm{I}}{\rm{I}}})=+1\end{array}$$

The cost ratio of the *minority* class against the majority class is *λ*_1_/*λ*_2_. This rescaling ratio is implemented to rebalance the classes such that the influence of each class in the learning process is in proportion to its cost. After rescaling, the influence of the minority class should be *λ*_1_/*λ*_2_ times of the influence of the majority class. In particular, when *λ*_1_/*λ*_2_ = 1, this is the class-balance learning. In medical diagnosis, the ratio is often larger than 1 because the mistakenly diagnosing a patient to be healthy may threaten a life.

According to the optimization theory^24^, the goal of prediction model can be written in the form of

5 $${{\rm{M}}{\rm{a}}{\rm{x}}}_{\pi }\,{{\rm{E}}}_{{\rm{Q}}}[{\rm{\Pi }}(\pi ({q}_{s}({t}_{{\rm{I}}}))={c}_{s}({t}_{{\rm{I}}{\rm{I}}})|\pi )]$$

6 $${\rm{s}}.{\rm{t}}.\{\begin{array}{c}{\lambda }_{1}({\rm{\Pi }}(\pi ({q}_{s}({t}_{{\rm{I}}}))\ne {c}_{s}({t}_{{\rm{I}}{\rm{I}}})|{c}_{s}=-1)+{\lambda }_{2}({\rm{\Pi }}(\pi ({q}_{s}({t}_{{\rm{I}}}))\ne {c}_{s}({t}_{{\rm{I}}{\rm{I}}})|{c}_{s}=+1)\le {{\rm{C}}}_{{\rm{h}}{\rm{a}}{\rm{r}}{\rm{d}}}\\ {q}_{s}({t}_{{\rm{I}}})\in Q\end{array}$$

where C_hard_ is determined by domain experts. The tuition of this prediction model is to maximize the accuracy of learner *π* on the constraint of a given cost bound C_hard_.

Here we presents a variant of ensemble learning methods^23^, cost-sensitive hybrid method (CSHM), which has the advantage of being able to covert weak learners to strong learners. To exploit the independence between the base learners, the weak learners are combined in a parallel way to improve their performance. Each base predictor has an independent generalization accuracy, i.e., for base classifier *π*_*l*_, *Acc* (*π*_*l*_) = E_*x*∼*D*_[Π(*π*_*l*_ (*x*) = *f*(*x*))]. sign(·) is a signal function. After combing *L* number of such base predictors according to

7 $$\pi ({q}_{s}({t}_{{\rm{I}}}))=sign(\frac{1}{L}\sum _{l=1}^{L}{\pi }_{l}(x))$$

this hybrid prediction model *π* makes an error only when at least half of its base predictors make errors. In practice, the heterogeneous base predictors can be selected from conventional machine learning algorithms.

### Evaluation Design

Our prediction framework mainly contained classification models with embedded feature selection methods, which adaptively found the optimal feature set from the raw EHRs data for each classification model. Before we obtained the whole cohort data set, we primarily extracted a balanced data set from the data repository as an example to select the methods. In the experiments, we conducted 10-fold cross validation based on each model-based predictor. During the training period, we firstly employed and compared six widely-used classification models^25^ to identify the most feasible machine learning (ML)-based prediction technique. Those base learners had good performance in predicting the positives. However, the whole data set was found imbalanced after completing the whole process of data cleaning. Then, to measure the effectiveness of prediction, we had to implement it in a variety of decision costs with the imbalanced data. The baseline classifiers used here contained Logistic Regression (LR), Bayesian network (BN), Neural network (NN), support vector machine (SVM), and CHAID tree. Systematic and comprehensive benchmark of different machine learning models was beyond the scope of this paper. To keep our work focused and data-efficient, we adapted parameters and decision thresholds of those predicted models^25^, as shown in Table 2. In particular, we used 0.50 as the classification threshold for the classifiers LR and NN. Based on the selected base classifiers, CSHM and its five variants with different decision costs were implemented to model patterns of GDM samples and normal ones. According to the domain experts, the cost rate for the misprediction of the positive and negative samples could be set at a certain ratio (i.e., 100:1) while the costs of correct prediction zero.

**Table 2:** Setting details of the six methods.

Methods	Details of setting
Logistic Regression	Procedure: polynomial
	Selection of variables in equation fitting: forward
	Target class: 1, Model type: main effect
	Include constants in the equation
Bayesian network	Structure type: Markov cover
	Parameter learning method: maximum likelihood
Neural Networks	Primary objective: Enhanced model accuracy(boosting) Model: multilayer perceptron NN
	Hidden layer: automatically calculates the number of cells
	Termination rule: Maximum number of training cycles=250
	Number of component models (boosting):10
	Prevent over fitting sets: 30%
Support Vector Machines	Kernel: radial basis function (non-linear)
	Stop threshold: 1.0e-3
	Regression accuracy(epsilon): 0.1
CHAID trees	Tree growth algorithm: CHAID
	Maximum tree depth: 16Termination rule: ①Minimum number of records in a parent branch: 2.0%; ②The minimum number of records in a child branch: 1.0%
	Segmentation and merging: Significance level(0.05)
	Split Merge classes within a node: No
	The maximum number of iterations of convergence: 200
CSHM	Base classifiers: LR, SVM, CHAID trees Method: Confidence weighted voting (maximum)
	Model discard criteria: AUCROC < 0.6
	Cost ratio: *λ*_1_ = 1, 1.5, 5, 10, 100 and 1000.

During the testing period, we implemented the trained models to predict the labels of the new instances. Moreover, five measures^26,27^ including accuracy, area under the curve (AUC), true positive rate (TPR), false positive rate (FPR) and confidence report^28,29^ (mean correct and mean incorrect) were adapted to evaluate the performance of these classifiers. In particular, true positive rate (TPR) was implemented to measure the effectiveness of predicting positives.

### EHR Processing

- Discretization and normalization. The continuous attributes of the input data were converted into discrete ones (intervals). The ‘sweep’ function in ‘R’ software^30,31^ was also implemented for Max-Min normalization.
- Missing data processing. Rate of missing data reflected the randomness of missing data, because pregnant women received heterogeneous recommendations from the physicians. Besides, irregular examinations also took missing values. To make the experimental data fit for classification (i.e., no less than 20% of missing values in attributes and no less than 5% in samples), the missing values were automatically filled by the interpolation algorithm (knnImpute)^32^, in which its function mice was set as (data set, m = 5, meth = PMM).
- Selected features. With the above processed data set, the optimal features were obtained with the embedded feature selection methods^33^.

### Future GDM risk prediction

To predict the risk that gestational women might develop GDM given their current clinical status, we implemented cost-sensitive hybrid model trained over historical instances using our study dataset. This is because it often demonstrate better performances than other baseline classifiers. When records of new patients are acquired, her clinical characteristics will be handled with through the proposed data processing stream. Then, the future GDM risk of those new cases can be predicted as GDM or normal with the selected classifying model.

## Results

This paper implemented a data set of a pre-set time window to execute the prediction task. The model for early diagnosis of GDM by machine learning (ML) had the ability to predict the unknown GDM status of pregnant women in their early pregnancy by exploring the value of EHRs, including archival data, examination data and diagnostic data of OGTT. After filtering in the raw data set, there were 4,378 cases and 50 attributes in the experimental data. Among them, 3940 samples (90%) were used for training and the other 438 samples (10%) for testing. We configured these algorithms in a 10-fold cross validation way. We firstly employed and compared several widely-used classification models to identify the most feasible ML-based prediction technique during the training period. Then, to measure the effectiveness of the selected model with imbalanced data, we implemented it in a variety of contexts of decision costs. For each model-based predictor, we conducted cross validation^34^ with their embedded optimal feature sets. Through preliminary experiments on the validation dataset, the future risk to develop GDM was identified for each patient in this period.

### Comparisons of Predictive Algorithms in Discrimination Ability

**Figure 2:**
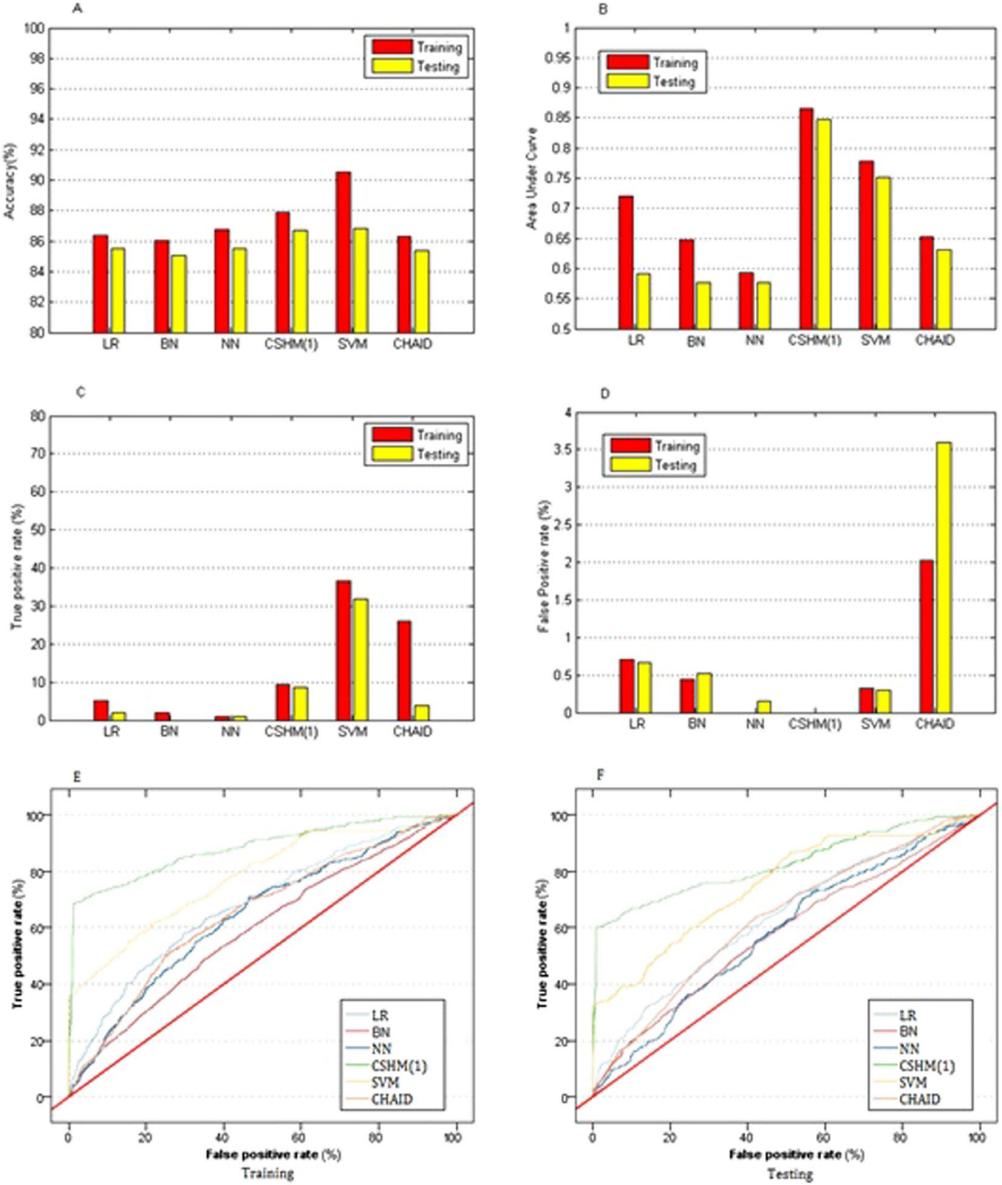
Performance of six techniques with cross validation. Bar graphs in (**A**), (**B**), (**C**) and (**D**) illustrate accuracy, area under ROC curve (AUC), true positive rate (TPR) and false positive rate (FPR) of those six techniques, respectively. Curves in (**E**) and (**F**) demonstrate receiver operating characteristic (ROC) for training and testing. LR: logistic regression; NB: naive Bayes; NN: neural network; SVM: support vector machine; CHAID: Chi-square automatic interaction detection Tree; CSHM (1): cost-sensitive hybrid model with cost parameter λ_1_=1 (symmetrical costs of misclassification). TPR and FPR are obtained from their confusion matrix.

To identify the most feasible algorithm for predicting GDM, we first employed and compared those six techniques with cross validation in their discrimination abilities, as shown in Fig. 2. The accuracies of Logistic Regression (LR), Bayesian network (BN), Neural network (NN), support vector machine (SVM), CHAID tree and a variant of ensemble methods (cost-sensitive hybrid model, CSHM (1)) in both training and testing were between 85.04% and 87.9%, except that the accuracy of SVM in training was over 90% (Fig. 2A). Although their accuracies were high, other measures were introduced to further compare their performance because the experimental data were imbalanced and almost 86% of the experimental instances were negative. In area under ROC curve (AUC s) (Fig. 2B), the data demonstrated that CSHM (*λ*_1_ = 1) was superior to LR, NB, NN, SVM and CHAID in both training and testing. AUC of CSHM (*λ*_1_ = 1) was 0.865 for the training and 0.847 for the test.

Despite of their accuracies and AUCs, true positive rate (TPR) and false positive rate (FPR) were also deduced from their confusion matrix to compare their performance (Fig. 2C and D). On the one hand, the TPRs of LR, NB and NN were very low, which were below 5% or even 0, while the TPRs of SVM were the highest among them in both training and testing. The TPRs of CSHM (*λ*_1_ = 1) were around 10% in training and testing and had less variance than those of SVM and CHAID, of which the TPRs had the largest variance. On the other hand, FPRs of those six techniques were very low, especially that FPRs of CSHM (*λ*_1_ = 1) were even 0. Those results depicted that those techniques did not or seldom predict the instances as positive, but once the instances were predicted as positives, those instances would be identified as real positives in OGTT at very high future risks. That is, we are less certain that a gestational woman does not have GDM if she has not been identified by those methods with EHRs at the first trimester, while we have a high confidence that a woman has GDM if she has been identified for the disease.

Furthermore, receiver operating characteristic (ROC) curves of those techniques demonstrated that CSHM was significantly better than the other five methods for training and testing (Fig. 2E and F). Those results depicted that although the discrimination abilities of positive samples were not high for those techniques without considering the imbalance of the data, the performance of CSHM (*λ*_1_ = 1) showed the best in those measures collectively.

### Effectiveness of CSHM with Asymmetrical Costs of Misclassification

**Figure 3:**
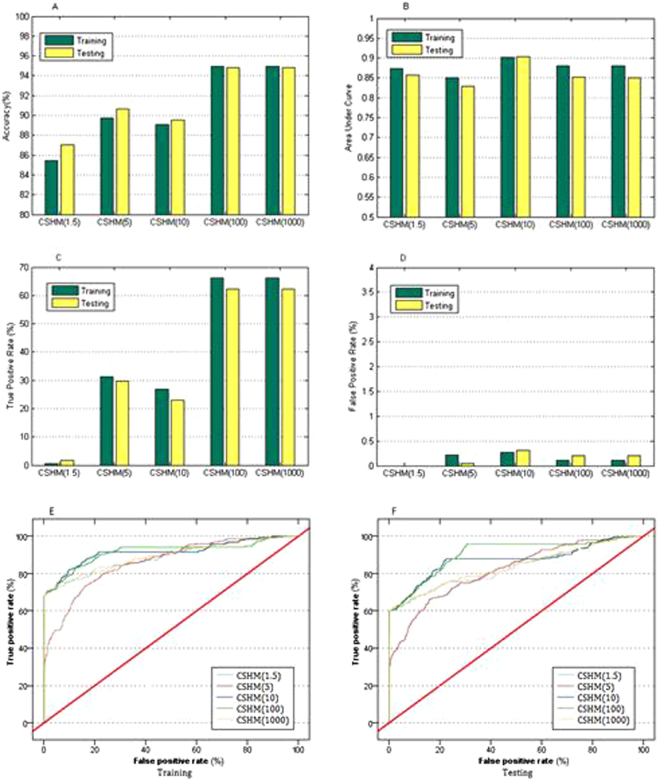
Performance of CSHM in five cost sensitive contexts with cross validation. Bar graphs in (**A**), (**B**), (**C**) and (**D**) illustrate accuracy, area under ROC curve (AUC), true positive rate (TPR) and false positive rate (FPR) of CSHM in five cost sensitive contexts, respectively. Curves in (**E**) and (**F**) demonstrate receiver operating characteristic (ROC) for training and testing. CSHM (1.5): cost-sensitive hybrid model with cost parameter λ_1_ = 1.5 (asymmetrical costs of misclassification). TPR and FPR are obtained from their confusion matrix.

Performance of CSHM with the parameter *λ*_1_ has also been verified by more experiments, because the values of *λ*_1_ reflect the preference of domain experts. With the experimental data, Fig. 3 shows the results of CSHM with certain decision costs.

From accuracy and AUC, the results (Fig. 3A and B) depicted that CSHM in sensitive contexts (*λ*_1_ = 1.5, 5, 10, 100 and 1000) presented good performance. When *λ*_1_ = 10, its AUC (Fig. 3B) achieved the peaks at 0.902 and 0.893 for training and testing. In accuracy (Fig. 3A), CSHM with *λ*_1_ = 100 or 1000 showed a better discrimination ability in learning than with other values, as well as TPRs (Fig. 3C). In FPR (Fig. 3D), all the variants of CSHM presented low values. Although the FPR of CSHM with *λ*_1_ = 1.5 was lower than those of the other cases, its TPR did not show any strength in discrimination ability of positive samples. The variances of TPRs of the variants were much larger than those of FPRs. In total, for imbalanced and cost-sensitive data, CSHM with a larger cost (i.e., 100) demonstrated more effective in discrimination ability than those with much smaller values (i.e., 1.5) (Fig. 3E and F).

Another relative method to solve the problem of imbalance learning was balanced sampling, such as undersampling^13,35^. It mainly consisted of two steps: trading off the balance of the positive samples and the negative with undersampling; training the new generated data set with machine learning algorithms. This kind of sampling method removed a part of the majority samples, while CSHM with asymmetrical costs did not here, which maximized the values of the acquired EHRs data and proved effective for identifying GDM.

### Significance Analysis

**Figure 4:**
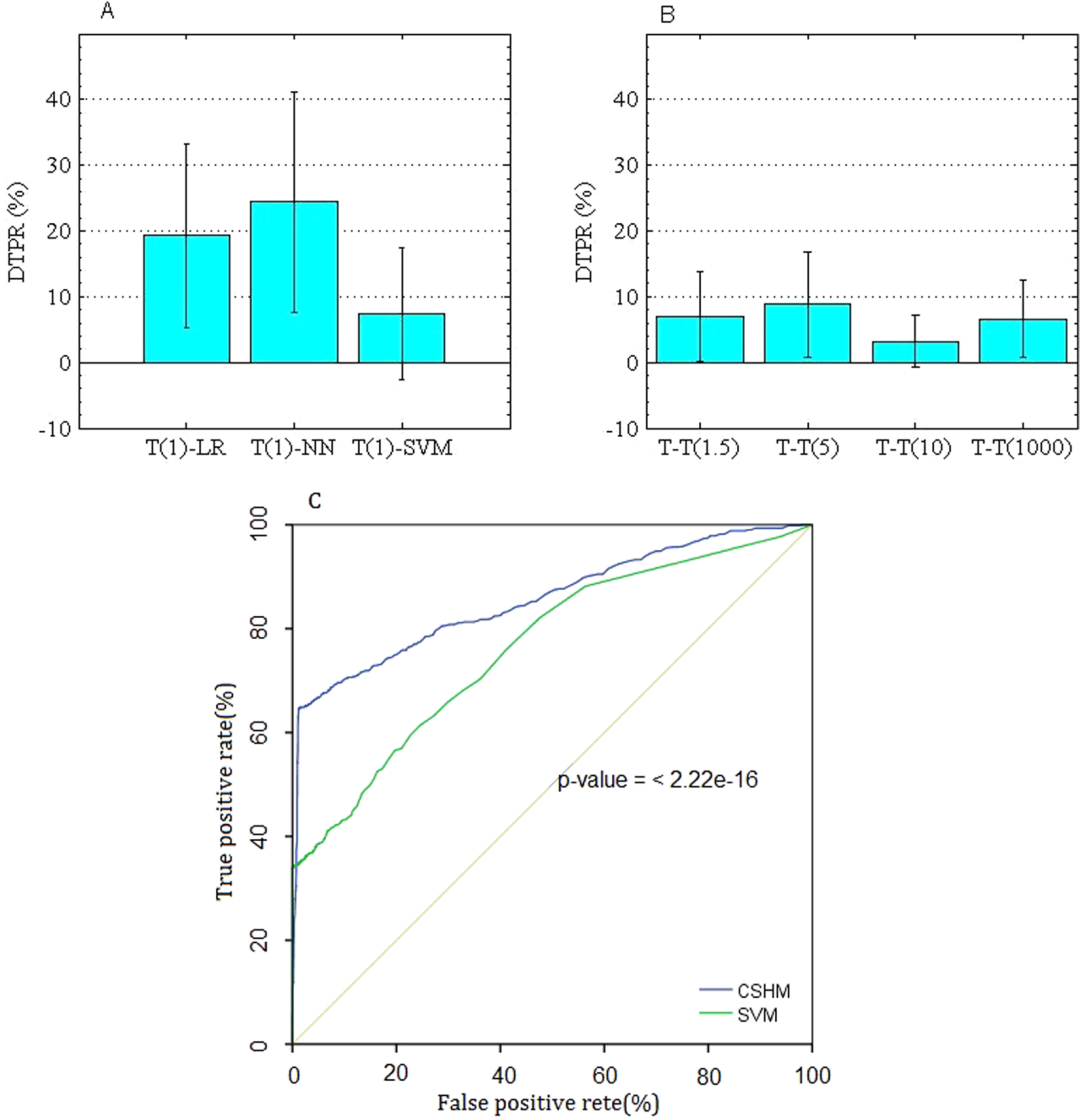
Significance of CSHM comparing with other methods. (**A**) Significance of CSHM to the algorithms of SVM, LR and NN; (**B**) significance of CSHM(100) to the other four cost sensitive contexts. (**C**) Comparison of the results with CSHM and SVM on the experimental data set. T(1): CSHM(1), CSHM model takes the cost parameter λ_1_=1. T(1)-LR (or NN, SVM): the true positive rates of CSHM(1) minus those of LR (or NN, SVM). T(100)-T(1)(or T(5), T(10), T(1000)): the true positive rates of CSHM(100) minus those of CSHM(1) (or T(5), T(10), T(1000)). p-value < 0.001 illustrates the significance of those two methods with a two-sided test for difference in AUC.

In the studies on permutation tests^36^, more statistical measures were used to validate the significance of AUC for each algorithm. Although we have compared ROC and AUC through 10-cross validation method, here we executed more experiments to compare the significance of true positive rates with certain thresholds of false positive rates. The significance of CSHM compared to the algorithms of SVM, LR and NN and its variants in five cost sensitive contexts were shown in Fig. 4(A) and (B). Here we introduced a new concept, difference in true positive rates (DTPR). Given N thresholds of false positive rates, DTPR is the difference of the true positive rates of model Y_1_ minus those of model Y_2_ at each threshold.

8 $${\rm{D}}{\rm{T}}{\rm{P}}{\rm{R}}({{\rm{Y}}}_{1}{\textstyle \text{-}}{{\rm{Y}}}_{2})\,=\,{\rm{T}}{\rm{P}}{\rm{R}}({{\rm{Y}}}_{1})\,-\,{\rm{T}}{\rm{P}}{\rm{R}}({{\rm{Y}}}_{2})$$

For example, the difference between CSHM and other methods is obtained through subtracting TPRs of SVM, LR and NN from those of CSHM, respectively.

First, we verified the significance of CSHM (1) to the algorithms of SVM, LR and NN. Then, we verified the significance of CSHM (100) to the other four cost sensitive contexts. In the experiments, we achieved the data sequence of true positive rates (TPR) at 1871 and 1887 thresholds of false positive rates for those two cases, respectively, as shown in Table 3. In one-sample T-Test method^37^, we set the test value as 0. The results showed that those means were all larger than 0 for DTPR (CSHM-LR), DTPR (CSHM-NN) and DTPR (CSHM-SVM). Similarly, those means were all larger than 0 for DTPR (CSHM(100)- CSHM(1)), DTPR (CSHM(100)- CSHM(5)), DTPR (CSHM(100)- CSHM(10))and DTPR (CSHM(100)- CSHM(1000)). The results in Table 3 show that for those methods p < 0.001, which means the null assumption is acceptable in significance of those data.

**Table 3:** Significance comparison.

	Abbreviation	N	Mean	standard deviation	Standard error of mean	t	degree of freedom	Sig.(Two-sided)	Lower bound*	Upper Bound*
CSHM-LR	T(1)-LR	1871	0.1926	0.13892	0.00321	59.961	1870	<0.001	0.1863	0.1989
CSHM-NN	T(1)-NN	1871	0.2439	0.16816	0.00389	62.727	1870	<0.001	0.2362	0.2515
CSHM-SVM	T(1)-SVM	1871	0.0733	0.10030	0.00232	31.623	1870	<0.001	0.0688	0.0779
CSHM(100)-CSHM(1)	T(100)- T(1)	1887	0.0698	0.06764	0.00156	44.828	1886	<0.001	0.0667	0.0729
CSHM(100)-CSHM(5)	T(100)- T(5)	1887	0.0880	0.08092	0.00186	47.218	1886	<0.001	0.0843	0.0916
CSHM(100)-CSHM(10)	T(100)- T(10)	1887	0.0320	0.03911	0.00090	35.562	1886	<0.001	0.0302	0.0338
CSHM(100)-CSHM(1000)	T(100)–T(1000)	1887	0.0665	0.05797	0.00133	49.811	1886	<0.001	0.0639	0.0691

*95% confidence interval of difference.

Furthermore, the significance of CSHM to the algorithm SVM was verified particularly, as shown in Fig. 4(C). The statistical comparison of two correlated ROCs^38^ has been executed by the pROC Package^39^. The study of SVM ensembles^40^ provided an evidence that the ensembles have better performance than SVM. On the experimental data, p-value < 0.001 illustrates CSHM outperforms SVM significantly with a two-sided test for difference in AUC.

### Confidence Analysis of Prediction

**Figure 5:**
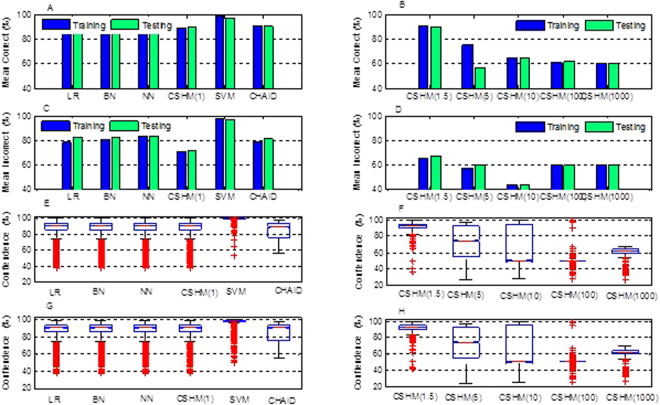
Confidence reports of six techniques and CSHM in five cost sensitive contexts with cross validation. Bar graphs in (**A**) and (**B**) illustrate mean correct and bar graphs in (**C**) and (**D**) illustrate mean incorrect of those six techniques and CSHM in five cost sensitive contexts, respectively. Boxplots in (**E**) and (**F**) illustrate confidence distributions for training and those in (**G**) and (**H**) illustrate confidence distributions for testing of those six techniques and CSHM in five cost sensitive contexts, respectively. Mean correct: mean confidence of correct predictions; mean incorrect: mean confidence of incorrect predictions.

Instead of black box modelling, it would be interesting for the medical community to know the confidence of those predictions that are being deduced by these classifiers in predicting GDM during early pregnancy. Figure 5 demonstrated the confidence reports of six techniques and the variants of CSHM in five cost sensitive contexts with cross validation. Comparing the confidence reports of CSHM (*λ*_1_ = 1) with those of LR, BN, NN, SVM and CHAID, the results demonstrated that the mean correct of CSHM was high with the lowest mean incorrect. It verified that the discrimination ability of CSHM was the best among those six methods. In details, the mean correct (Fig. 5A) of the training (test) set was 0.896 (0.897) for CSHM (*λ*_1_ = 1), indicating that the mean value of the prediction confidence of all correctly predicted samples was 0.896 (0.897). Its mean incorrect (Fig. 5C) of the training (test) set was 0.703 (0.718), indicating that the mean value of the prediction confidence for all error prediction samples was 0.703 (0.718). For training and testing, the confidence ranges of CSHM (*λ*_1_ = 1) were 0.374–0.997 (Fig. 5E) and 0.375–0.991 (Fig. 5G). Namely, there was no prediction with confidence below 0.374 (0.375) in the training (test) set. The results also showed that 1.92% of the observations in the training set were always higher than the confidence level 0.979. Similarly, 6.21% in the test set were always higher than the confidence level 0.655. For those samples with confidence over 0.604 (0.581) in the training (testing) set, more than 90.02% (90.04%) of the samples were correctly predicted with CSHM (*λ*_1_ = 1).

For the variants of CSHM with asymmetrical costs (*λ*_1_ = 1.5, 5, 10, 100 and 1000), the results in Fig. 5B demonstrated that the mean confidences of correct predictions were in a slope tread as *λ*_1_ grew for training. In details, the mean correct of CSHM with *λ*_1_ = 100 was 0.611 (0.615) for training (testing), indicating that the mean value of the prediction confidences was 0.611 (0.615) for all the correctly predicted samples. The mean confidence of CSHM (*λ*_1_ = 1.5) took the peak at 0.909, while that of CSHM (*λ*_1_ = 1000) took the lowest at 0.599. Meanwhile, CSHM (*λ*_1_ = 1.5) also took a smaller confidence range than those of the others (Fig. 5F). However, the mean confidence of incorrect predictions did not follow an obvious trend as *λ*_1_ grew (Fig. 5D). For example, the mean incorrect of the training (test) set was 0.601(0.6) for CSHM (*λ*_1_ = 100), indicating that the mean value of the prediction confidence was 0.601 (0.6) for all incorrectly predicted samples. The boxplots (Fig. 3F and H) illustrated that the distributions of confidence varied a lot among the variants of CSHM with different sensitive costs. As *λ*_1_ increased, both of the upper and lower bounds of the confidence ranges were in decreasing trends. The confidence of CSHM (*λ*_1_ = 1.5) ranged from 0.395 to 0.996 in training, while that of CSHM (*λ*_1_ = 1000) ranged from 0.264 to 0.666. Those patterns were also different from other relative methods (including LR, BN, NN, SVM and CHAID). In short, those results suggested that the confidences of those prediction methods were sensitive to the decision costs of the imbalanced data set.

## Discussion

This study which has been conducted in a Chinese population in West China Second Hospital, highlights some novel and potentially clinically important aspects of routine and nonroutine tests to predict GDM. Although several machine learning techniques with a panel of maternal demographic and clinical characteristics in EHRs may dependently predict the risk for GDM, the results show that the ensemble method CSHM with asymmetrical costs of misclassification provides better predictive ability. The tuition of prediction is: after the attributes being extracted and selected from the EHRs historical data, the machine learning models are employed and trained with two subsets (GDM and non-GDM); then, implicit temporal patterns are achieved by those models from those characteristics data; finally, for a new (undiagnosed) pregnant women, the instance of her record is input into the selected and trained model and the occurrence probability of GDM is deduced as its future risk.

Our work has a number of strengths. First, the possibility of early screening is advanced by our technique and data. With EHRs high dimensional data, our data give a better overall reflection of prediction of GDM in women without prior GDM at the first trimester. The clinical utility of EHRs in the first trimester is enhanced by virtue of not being altered in nonfasting samples (unlike OGTT)^7^. Their applications aid the physicians to distinguish the high risk of GDM candidates from the gestation women at the first trimester which is much earlier than OGTT period. Those women also benefit from the predictive insights. The prediction results of GDM will caution those gestational women with high future risks and provide an important way to enhance their health.

Second, we identify CSHM as the most feasible algorithm from those six machine learning models for predicting GDM. In general, those six machine learning models^25^ are available to predict GDM in early pregnancy, while the performance of CSHM model shows the best in the experiment. CSHM presents high sensitivity and low false positive rate, illustrating better in predicting positive instances than the other five relevant prediction techniques. Although the accuracy of positive samples is barely acceptable (62.16%), the prediction accuracy of negative samples is high (99.8%). Among those predicted positive instances, the results suggest that the vast majority (98.4%) are real GDM class according to OGTT. Our results also suggest that although CSHM takes lower confidence in prediction than a simple classifier, it is very good at prediction of GDM with higher AUC than those of the others.

Finally, our work is different from prospective cohort studies on GDM prediction, and our results suggest that further development and potential clinical application of risk algorithms for GDM in a range of populations is possible. In cohort studies on GDM, their methods require balanced panel data and the sizes of their data set are limited. For instance, Savvidou, M. *et al*.^14^ just investigated only 124 and 248 mixed ethnic population cases recorded as GDM and control subjects in their study, although yielding an AUC of 0.861 for GDM. In our study, those maternal information in EHRs are readily accessible and these feature data are available in most women and children’s hospitals in China. Furthermore, we were very careful in the maternal data during the experiment and considered relevant missing values and cost-sensitive models for consistency of findings.

We acknowledge a number of limitations. For all the six prediction techniques based on supervised machine learning algorithms, their input features are extracted and selected from the pre-processed EHRs in an ad-hoc fashion. Since the predictive algorithms have the embed function of feature selection^33^, risk factors haven’t been investigated in our analyses. To make the attributes in the models easily interpretable for the physicians, the framework would be paired with a feature selection tool to help the clinicians understanding what drove the different predictions. Furthermore, we acknowledge the lack of detailed validation of our models in other data or the lack of follow up the positive cohorts of the validation. Our aim is not to define new insights on the risk factors but rather to prompt others to advance our findings toward possible clinical utilities.

Collectively, the results demonstrate that risks for GDM can be predicted in the first trimester of pregnancy from a mix of maternal demographic and characteristics. Our study should also encourage others to test and validate similar ML-based prediction techniques for GDM in the same way. The possibility of first-trimester identification of women at greatest risk of GDM, with subsequent implementation of possible lifestyle or medical interventions at this stage, requires further study. The method used herein is effective to the imbalanced clinical data, in which the resampling method may lead to other problems. For instance, oversampling methods may lead to class distribution shift when running too many iterations, undersampling methods may lead to samples (and their implied knowledge) missing. In the future, to improve the performance of the prediction method, we will try those resampling methods and comparing them with the CSHM, even combining manipulations at the data-level with classifier-level modifications. To clarify the significant features in predicting GDM, more investigation will be explored to find the optimal set of input features by integrating the domain knowledge of medical experts and the attributes of those models.

## Electronic supplementary material

Supplementary Information
