# Supplementary material for: Electronic Health Record Driven Prediction for Gestational Diabetes Mellitus in Early Pregnancy
Source: Sci Rep. 2017 Nov 27;7:16417. doi: 10.1038/s41598-017-16665-y (PMC5703904; doi:10.1038/s41598-017-16665-y)
Supplement: Supplementary file 1 — Supplementary Information [file 41598_2017_16665_MOESM1_ESM.pdf]

Electronic Health Record Driven Prediction for Gestational Diabetes Mellitus in Early Pregnancy

Hang Qiu[1,2*], Hai-Yan Yu[1,2,3,4*], Li-Ya Wang[1], Qiang Yao[5], Si-Nan Wu[6], Can Yin[7], Bo Fu[1,2], Xiao-Juan Zhu[1,2], Yan-Long Zhang[7], Yong Xing[7], Jun Deng[7], Hao Yang[8], Shun-Dong Lei[6]

[1]Big Data Research Center, University of Electronic Science and Technology of China, Chengdu, 611731, Sichuan, China. [2]School of Computer Science and Engineering, University of Electronic Science and Technology of China, Chengdu, 611731, Sichuan, China. [3]School of Economics and Management, Chongqing University of Posts and Telecommunications, Chongqing, 400065, Chongqing, China. [4]Department of Statistics, The Pennsylvania State University, University Park, PA 16802-2111, United States. [5]Division of Obstetrics, West China Second University Hospital, Sichuan University, Chengdu, 610041, Sichuan, China. [6]Division of Information Management, West China Second University Hospital, Sichuan University, Chengdu, 610041, Sichuan, China. [7]Chengdu Shulianyikang Technology Co., Ltd, Chengdu, 610041, Sichuan, China. [8]School of Computer Science, Chengdu University of Information Technology, Chengdu, 610225, Sichuan, China.

[*]These authors contributed equally to this work. Correspondence and requests for materials should be addressed to H.Y.Y (email: yhy188@gmail.com) or S.D.L (tomlsd@163.com)

Appendix A1.

Let us consider a two-class prediction problem (binary classification), in which the outcomes are labeled either as positive (p) or negative (n). There are four possible outcomes from a binary classifier. If the outcome from a prediction is p and the actual value is also p, then it is called a true positive (TP); however if the actual value is n then it is said to be a false positive (FP). Conversely, a true negative (TN) has occurred when both the prediction outcome and the actual value are n, and false negative (FN) is when the prediction outcome is n while the actual value is p. We adapted six measures[3,4] to evaluate the performance of the classifiers, including:

①Accuracy, Acc(%)=(TP+TN)/(TP+FN+FP+TN)

②AUC denotes the Area Under the Curve, which is equal to the probability that a classifier will rank a randomly chosen positive instance higher than a randomly chosen negative one.

③TPR(%)=TP/(TP+FN), which defines how many correct positive results occur among all positive samples available during the test.

④FPR(%)=FP/(TN+FP), which defines how many incorrect positive results occur among all negative samples available during the test.

⑤Precision, P(%)=TP/(TP+FP), which is the fraction of retrieved instances that are relevant.

⑥Confidence Report[5,6]. Statistical report on confidence, including: Mean Correct: Mean Confidence of Correct Predictions; Mean Incorrect: Mean Confidence of Incorrect Predictions. Confidence Distribution has often been loosely referred to as a distribution function on the parameter space that can represent confidence intervals of all levels for a parameter of interest.

Appendix A2.

To make machine learning methods scalable and robust, we convert the input data sets with continuous attributes into input data sets with discrete attributes. The original continuous attributes are discretized into intervals. For BMI, there is a reference[7] standard for discretization. Although there were no rules for the discretization of "7 groups of age, and 8 groups of height, 5 groups of marriage years", those data was pre-processed as a normal procedure of data mining.

Table A1. Discretization rules of the continuous variables

Characteristic	values	Demonstration	GDM	Normal
BMI	1	<18.5	72	670
	2	≥18.5, ≤23.9	411	2687
	3	>23.9, ≤27.9	107	374
	4	>27.9	19	43
Age	1	<25	8(2)	164(74)
	2	≥25, <27	32(24)	358(237)
	3	≥27, <29	101(78)	820(626)
	4	≥29, <31	110(92)	812(691)
	5	≥31, <33	116(103)	605(681)
	6	≥33, <35	97(90)	479(491)
	7	≥35	144(219)	538(948)
Height	1	<140	0	1
	2	≥140, <145	1	1
	3	≥145, <150	2	12
	4	≥150, <155	63	324
	5	≥155, <160	215	1227
	6	≥160, <165	227	1451
	7	≥165, <170	90	622
	8	≥170	11	127
marriage_ages(Years)	1	[0, 3）	229	1791
	2	[3, 6）	176	947
	3	[6, 9）	94	478
	4	[9, 12）	48	235
	5	12+	48	167

Appendix A3.

According to International Association of the Diabetes and Pregnancy Study Groups (IADPSG) guidelines[1,2], the screening and diagnosis of GDM can routinely be executed at the period of 24-28 weeks' gestation. Risk factors such as fasting plasma glucose (FPG), 1h blood glucose, 2h blood glucose of OGTT or FPG in the second trimester are the determinant risk factors in women to identify GDM.

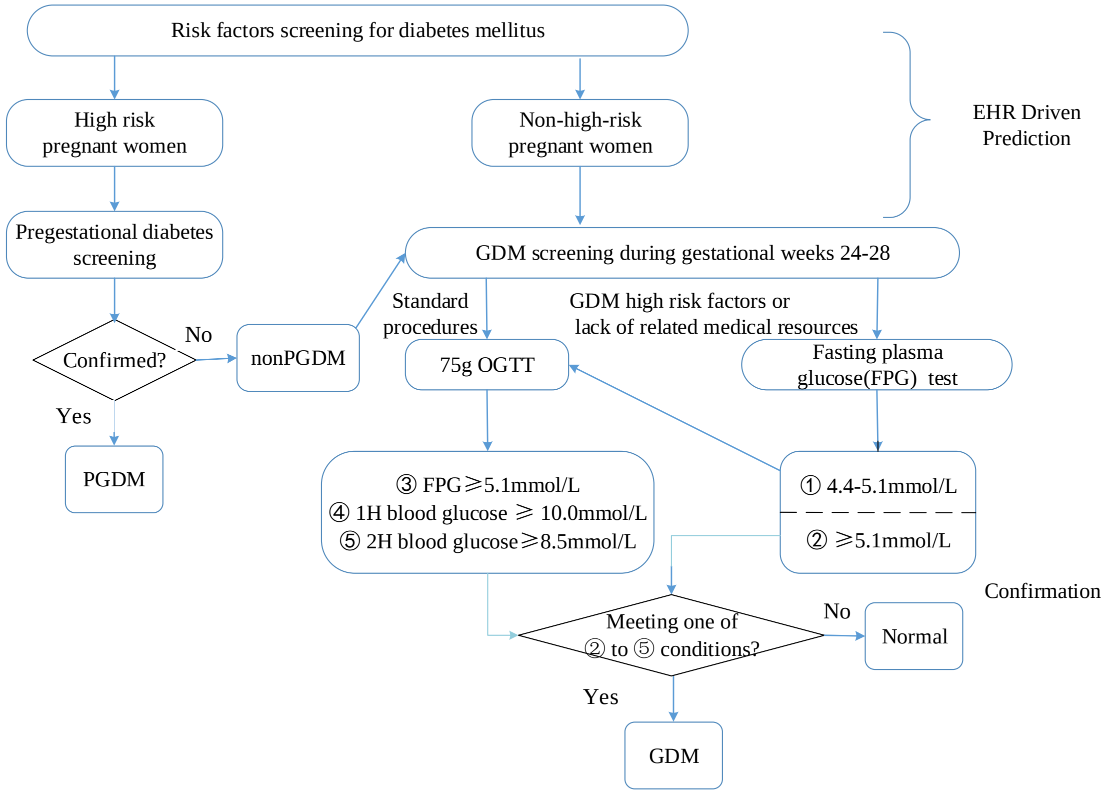

Figure A1. The screening and diagnosis guidelines for GDM in China.

Appendix A4.

The experimental data was obtained after the flitting and data cleaning process, as shown Figure **A2**.

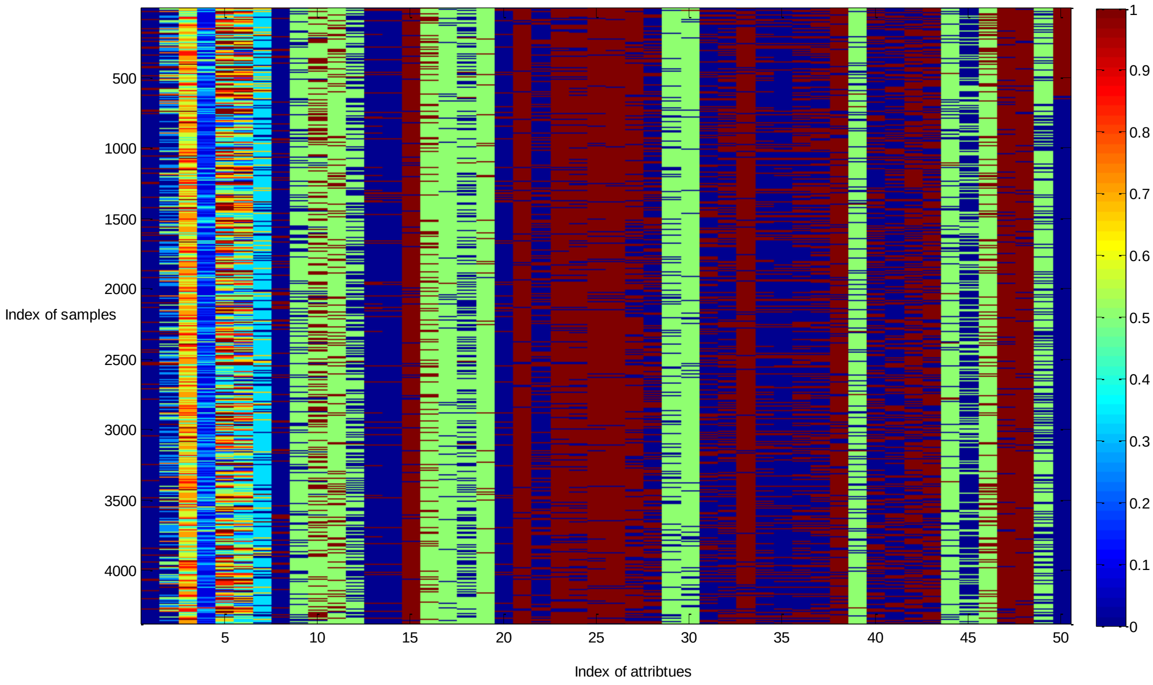

Figure A2. Data matrix of the experimental data after attributes normalization. Dark blue grids of the matrix represent 0, deep red grids represent 1.

Appendix A5.

For Logistic Regression (LR), the input attributes and their parameter estimates are demonstrated in table A2. Among those attributes, there are some redundancy.

Table A2 Input attributes and their parameter estimates of Logistic Regression (LR)

							95% Confidence Interval for Exp(B)	
label_gdm[a]	B	Std. Error	Wald	df	Sig.	Exp(B)	Lower Bound	Upper Bound
Intercept	-15.429	493.650	.001	1	0.975			
[high_risk=0]	0.075	0.332	0.052	1	0.820	1.078	0.563	2.065
[high_risk=10]	0[b]	0.	0.	0	0.	.	0.	.
[marriage_ages=0]	0.082	0.331	0.061	1	0.805	1.085	0.567	2.076
[marriage_ages=0.250]	0.318	0.320	0.986	1	0.321	1.374	0.734	2.572
[marriage_ages=0.500]	-0.067	0.319	0.044	1	0.834	0.935	0.501	1.748
[marriage_ages=0.750]	-0.274	0.330	0.691	1	0.406	0.760	0.398	1.451
[marriage_ages=10]	0[b]	.	.	0	.	.	.	.
[height=0]	-9.486	246.821	.001	1	.969	7.591E-5	6.106E-215	9.436E+205
[height=0.143]	1.178	1.508	.610	1	.435	3.246	0.169	62.332
[height=0.286]	1.876	1.023	3.363	1	.067	6.528	0.879	48.489
[height=0.429]	0.468	0.464	1.019	1	0.313	1.597	0.643	3.964
[height=0.571]	0.344	0.432	.634	1	0.426	1.410	0.605	3.286
[height=0.714]	0.269	0.430	.392	1	0.532	1.308	0.564	3.037
[height=0.857]	0.073	0.452	.026	1	0.872	1.076	0.443	2.610
[height=10]	0[b]	.	.	0	.	.	.	.
[pregnancy_times=0]	0.881	267.470	0.000	1	0.997	2.412	5.144E-228	1.131E+228
[pregnancy_times=0.083]	9.629	246.820	0.002	1	0.969	15199.999	1.225E-206	1.886E+214
[pregnancy_times=0.167]	9.610	246.820	0.002	1	0.969	14908.289	1.201E-206	1.850E+214
[pregnancy_times=0.250]	9.623	246.820	0.002	1	0.969	15114.515	1.218E-206	1.876E+214
[pregnancy_times=0.333]	9.478	246.820	0.001	1	0.969	13075.138	1.053E-206	1.623E+214
[pregnancy_times=0.417]	10.266	246.820	0.002	1	0.967	28730.918	2.314E-206	3.567E+214
[pregnancy_times=0.500]	9.317	246.821	0.001	1	0.970	11125.686	8.947E-207	1.384E+214
[pregnancy_times=0.583]	9.275	246.822	0.001	1	0.970	10672.120	8.577E-207	1.328E+214
[pregnancy_times=0.667]	.095	349.057	0.000	1	1.000	1.100	8.389E-298	1.441E+297
[pregnancy_times=0.750]	.528	280.941	0.000	1	0.999	1.695	1.235E-239	2.325E+239
[pregnancy_times=0.833]	11.450	246.825	0.002	1	0.963	93908.514	7.497E-206	1.176E+215
[pregnancy_times=0.917]	.372	349.057	0.000	1	0.999	1.451	1.107E-297	1.903E+297
[pregnancy_times=10]	0[b]	.	.	0	.	.	.	.
[husband_age=0]	-1.466	1.111	1.741	1	0.187	.231	0.026	2.037
[husband_age=0.167]	0.082	0.395	0.043	1	0.836	1.085	0.500	2.355
[husband_age=0.333]	-0.092	0.294	0.099	1	0.754	0.912	0.512	1.624
[husband_age=0.500]	-0.111	0.260	0.182	1	0.670	0.895	0.538	1.490

[husband_age=0.667]	-0.043	0.240	0.032	1	0.858	0.958	0.599	1.533
[husband_age=0.833]	-0.026	0.234	0.012	1	0.913	0.975	0.616	1.542
[husband_age=10]	0^b	0.	.	0	0.	0.	0.	.
[delivery_age=0]	-1.076	0.618	3.037	1	0.081	0.341	0.102	1.144
[delivery_age=0.167]	-1.321	0.483	7.495	1	0.006	0.267	0.104	0.687
[delivery_age=0.333]	-.965	0.395	5.949	1	0.015	0.381	0.176	0.827
[delivery_age=0.500]	-1.083	0.382	8.018	1	0.005	0.339	0.160	0.717
[delivery_age=0.667]	-.681	0.359	3.592	1	0.058	0.506	0.251	1.023
[delivery_age=0.833]	-.871	0.354	6.057	1	0.014	0.419	0.209	0.837
[delivery_age=10]	0^b	0.	.	0	0.	0.	0.	0.
[bmi=0]	-1.307	0.448	8.519	1	0.004	0.271	0.113	0.651
[bmi=0.333]	-1.170	0.410	8.151	1	0.004	0.310	0.139	0.693
[bmi=0.667]	-0.751	0.434	2.996	1	0.083	0.472	0.202	1.105
[bmi=10]	0^b	0.	.	0	.	.	0.	.
[Nonnative=0]	.261	0.231	1.281	1	.258	1.298	0.826	2.041
[Nonnative=10]	0^b	.	.	0	.	.	.	.
[HCT=0]	9.682	246.820	0.002	1	0.969	16023.330	1.292E-206	1.988E+214
[HCT=0.500]	9.414	246.820	0.001	1	0.970	12261.848	9.885E-207	1.521E+214
[HCT=10]	0^b	.	.	0	.	.	.	.
[MCH=0]	-0.493	0.449	1.206	1	0.272	0.611	0.253	1.473
[MCH=0.500]	0.192	0.155	1.540	1	0.215	1.211	0.895	1.640
[MCH=10]	0^b	.	.	0	0.	.	.	.
[WBC=0]	-9.066	159.307	0.003	1	0.955	0.000	2.889E-140	4.622E+131
[WBC=0.500]	-0.018	0.273	0.004	1	0.949	0.983	0.576	1.677
[WBC=10]	0^b	.	0.	0	0.	0.	0.	.
[EOS=0]	-1.236	1.371	0.813	1	0.367	0.291	0.020	4.265
[EOS=0.500]	-1.118	1.353	0.683	1	0.408	0.327	0.023	4.632
[EOS=10]	0^b	.	.	0	0.	.	.	.
[MPV=0]	1.713	0.634	7.299	1	0.007	5.548	1.601	19.228
[MPV=10]	0^b	.	.	0	0.	.	.	.
[PDW=0]	-0.237	0.458	00.267	1	0.605	0.789	0.321	1.938
[PDW=10]	0^b	.	.	0	0.	.	.	.
[RDW.CV=0]	0.539	0.288	3.514	1	0.061	1.714	0.976	3.012
[RDW.CV=10]	0^b	.	.	0	0.	.	.	.
[RDW.SD=0]	-10.626	169.669	0.004	1	0.950	2.428E-5	9.176E-150	6.426E+139
[RDW.SD=0.500]	0.043	0.197	0.047	1	0.829	1.044	0.709	1.537
[RDW.SD=10]	0^b	.	0.	0	0.	.	.	.
[MONO.=0]	-0.667	1.153	0.334	1	0.563	0.513	0.054	4.922
[MONO.=0.500]	-0.442	1.123	0.155	1	0.694	0.643	0.071	5.807
[MONO.=10]	0^b	.	0.	0	0.	.	.	.
[EOS.=0]	0.116	1.344	0.007	1	0.931	1.122	0.081	15.627
[EOS.=0.500]	0.479	1.323	0.131	1	0.717	1.614	0.121	21.593
[EOS.=10]	0^b	.	.	0	.	.	.	.

[PCT=0]	.451	1.192	.143	1	.705	1.569	.152	16.238
[PCT=0.500]	.409	.321	1.620	1	.203	1.505	.802	2.825
[PCT=10]	0[b]	.	.	0	.	.	.	.
[P.LCR=0]	-1.286	.557	5.327	1	0.021	0.276	0.093	0.824
[P.LCR=10]	0[b]	.	.	0	.	.	.	.
[HBsAg=0]	.086	0.349	0.060	1	0.806	1.089	0.550	2.158
[HBsAg=10]	0[b]	.	.	0	0.	.	0.	.
[Anti.HBs=0]	-.018	0.178	0.010	1	0.921	0.982	0.693	1.393
[Anti.HBs=10]	0[b]	.	.	0	0.	.	0.	.
[Anti.HBe=0]	.027	0.259	0.011	1	0.917	1.027	0.618	1.707
[Anti.HBe=10]	0[b]	.	.	0	0.	.	0.	.
[HBcAb.T.=0]	.206	0.229	0.803	1	0.370	1.228	0.784	1.925
[HBcAb.T.=10]	0[b]	.	.	0	0.	.	0.	.
[ALT=0]	-.586	0.386	2.305	1	0.129	0.557	0.261	1.186
[ALT=10]	0[b]	.	.	0	0.	.	0.	.
[AST=0]	.299	0.427	.493	1	0.483	1.349	0.585	3.113
[AST=10]	0[b]	.	.	0	0.	.	0.	.
[PA=0]	-.523	0.173	9.187	1	0.002	0.593	0.422	0.831
[PA=10]	0[b]	.	.	0	0.	.	0.	.
[UN=0]	.087	0.137	0.402	1	0.526	1.091	0.834	1.426
[UN=10]	0[b]	.	.	0	0.	.	.	.
[UA=0]	8.176	246.820	.001	1	0.974	3554.117	2.864E-207	4.411E+213
[UA=0.500]	8.307	246.820	.001	1	0.973	4051.883	3.265E-207	5.028E+213
[UA=10]	0[b]	.	.	0	0.	.	.	.
[FPG=0]	-15.793	0.248	4062.005	1	0.000	1.383E-7	8.512E-8	2.249E-7
[FPG=0.500]	-15.711	0.000	.	1	0.	1.502E-7	1.502E-7	1.502E-7
[FPG=10]	0[b]	0.	.	0	0.	.	.	.
[RBC=0]	-0.010	0.145	0.005	1	0.945	0.990	0.745	1.315
[RBC=10]	0[b]	0.	0.	0	0.	0.	0.	.
[EC=0]	-0.126	0.397	0.101	1	0.750	0.881	0.405	1.919
[EC=10]	0[b]	0.	0.	0	0.	.	.	.
[XYSPXB=0]	0.001	0.231	0.000	1	0.998	1.001	0.636	1.575
[XYSPXB=10]	0[b]	0.	0.	0	0.	.	0.	.
[CAST=0]	0.111	0.224	0.246	1	0.620	1.117	0.721	1.732
[CAST=10]	0[b]	0.	0.	0	0.	.	0.	.
[CAST.1=0]	-0.066	0.250	0.070	1	0.791	0.936	0.573	1.529
[CAST.1=10]	0[b]	0.	0.	0	0.	.	0.	.
[EC.1=0]	0.300	0.402	0.559	1	0.455	1.350	0.615	2.967
[EC.1=10]	0[b]	0.	0.	0	0.	.	0.	.
[WBC.1=0]	-0.130	0.167	0.613	1	0.434	0.878	0.633	1.216
[WBC.1=10]	0[b]	0.	0.	0	0.	.	0.	.
[TPOAb=0]	0.055	0.171	0.103	1	0.748	1.056	0.756	1.476
[TPOAb=10]	0[b]	.	0.	0	.	.	.	.

[TSH3UL=0]	-.232	0.550	0.177	1	0.674	0.793	0.270	2.333
[TSH3UL=0.500]	-.100	0.533	0.035	1	0.851	0.905	0.319	2.570
[TSH3UL=10]	0[b]	.	.	0	.	.	0.	.
[Anti.A=0]	-0.913	1.426	0.410	1	0.522	0.401	0.025	6.565
[Anti.A=10]	0[b]	.	0.	0	0.	0.	0.	.
[Anti.B=0]	-0.729	1.290	0.320	1	0.572	0.482	0.038	6.047
[Anti.B=10]	0[b]	.	0.	0	0.	0.	0.	.
[A1cells=0]	-0.764	1.425	0.288	1	0.592	0.466	0.029	7.604
[A1cells=10]	0[b]	.	0.	0	0.	0.	0.	.
[Bcells=0]	-0.607	1.289	0.221	1	0.638	0.545	0.044	6.825
[Bcells=10]	0[b]	0.	.	0	0.	0.	0.	.
[RBC.1=0]	-.675	0.611	1.220	1	0.269	0.509	0.154	1.687
[RBC.1=0.500]	-0.514	0.529	0.943	1	0.331	0.598	0.212	1.687
[RBC.1=10]	0[b]	.	.	0	0.	.	.	.
[LYMPH.=0]	9.394	246.820	.001	1	0.970	12015.003	9.682E-207	1.491E+214
[LYMPH.=0.500]	9.222	246.820	.001	1	0.970	10122.081	8.158E-207	1.256E+214
[LYMPH.=10]	0[b]	.	.	0	0.	.	.	.
[NEUT=0.500]	-0.422	0.259	2.658	1	0.103	0.656	0.395	1.089
[NEUT=10]	0[b]	0.	.	0	0.	0.	0.	.
[NEUT.=0]	-0.293	0.284	1.064	1	0.302	0.746	0.427	1.302
[NEUT.=10]	0[b]	.	.	0	0.	.	0.	.
[r.GT=0]	0.253	0.263	0.928	1	0.335	1.288	0.769	2.157
[r.GT=10]	0[b]	.	.	0	0.	.	0.	.
[ALP=0]	-3.371	1.555	4.700	1	0.030	0.034	0.002	.724
[ALP=0.500]	-3.001	1.548	3.758	1	0.053	0.050	0.002	1.034
[ALP=10]	0[b]	.	.	0	.	.	.	.

a. The reference category is: 1.0.

b. This parameter is set to zero because it is redundant.

Appendix A6.

For support vector machine (SVM), the input attributes and their parameter estimates are demonstrated in Figure A3(A). Among those attributes, there are some redundancy. We also demonstrate those results for CHAID tree, Bayesian network and Neural network (NN) in Figure A3(B) to Figure A3(D). For CSHM, the model takes all the attributes as the input data because those base classifiers select the attributes by their embedded methods. Although the parameter estimates of the input attributes are the same, they are also demonstrated in Figure A3(E). Among those attributes, all of their weights are near to 0.02.

A

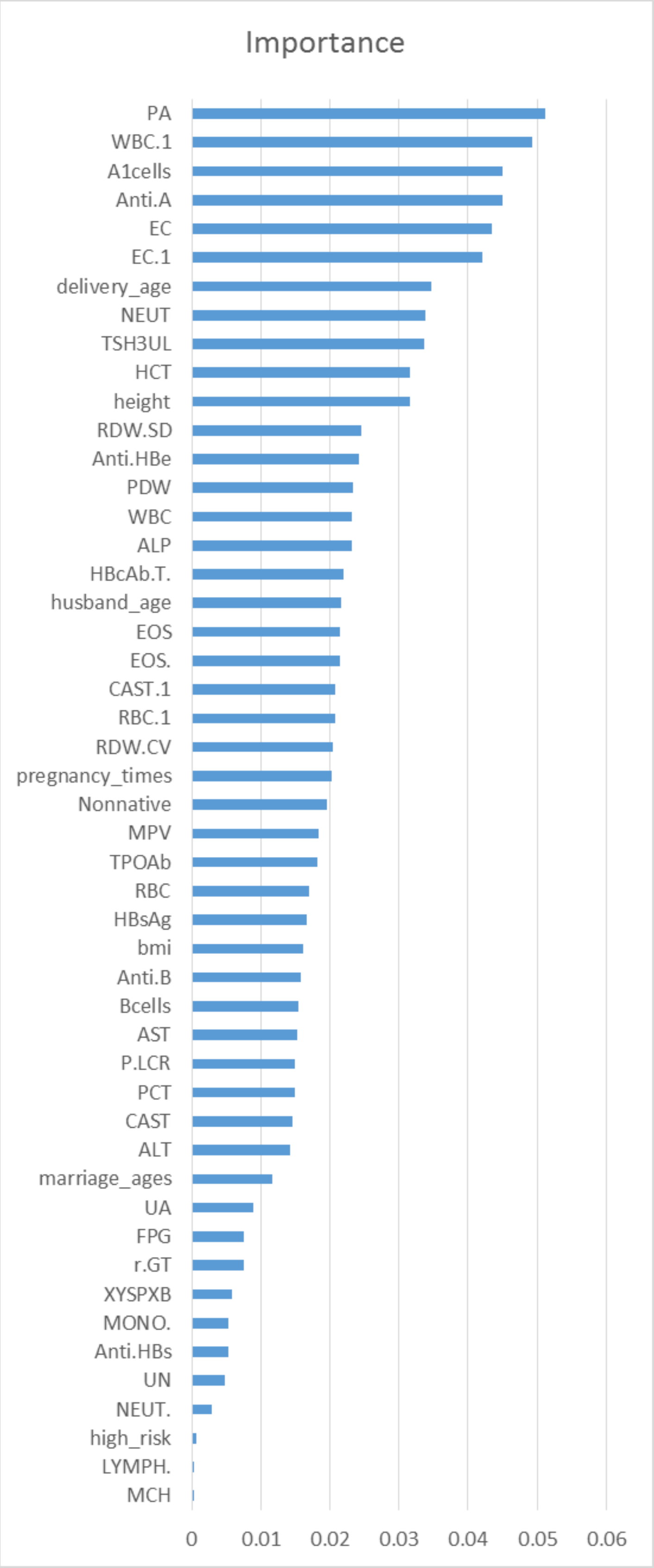
Importance
PA
WBC.1
A1cells
Anti.A
EC
EC.1
delivery_age
NEUT
TSH3UL
HCT
height
RDW.SD
Anti.HBe
PDW
WBC
ALP
HBcAb.T.
husband_age
EOS
EOS.
CAST.1
RBC.1
RDW.CV
pregnancy_times
Nonnative
MPV
TPOAb
RBC
HBsAg
bmi
Anti.B
Bcells
AST
P.LCR
PCT
CAST
ALT
marriage_ages
UA
FPG
r.GT
XYSPXB
MONO.
Anti.HBs
UN
NEUT.
high_risk
LYMPH.
MCH
0
0.01
0.02
0.03
0.04
0.05
0.06

B

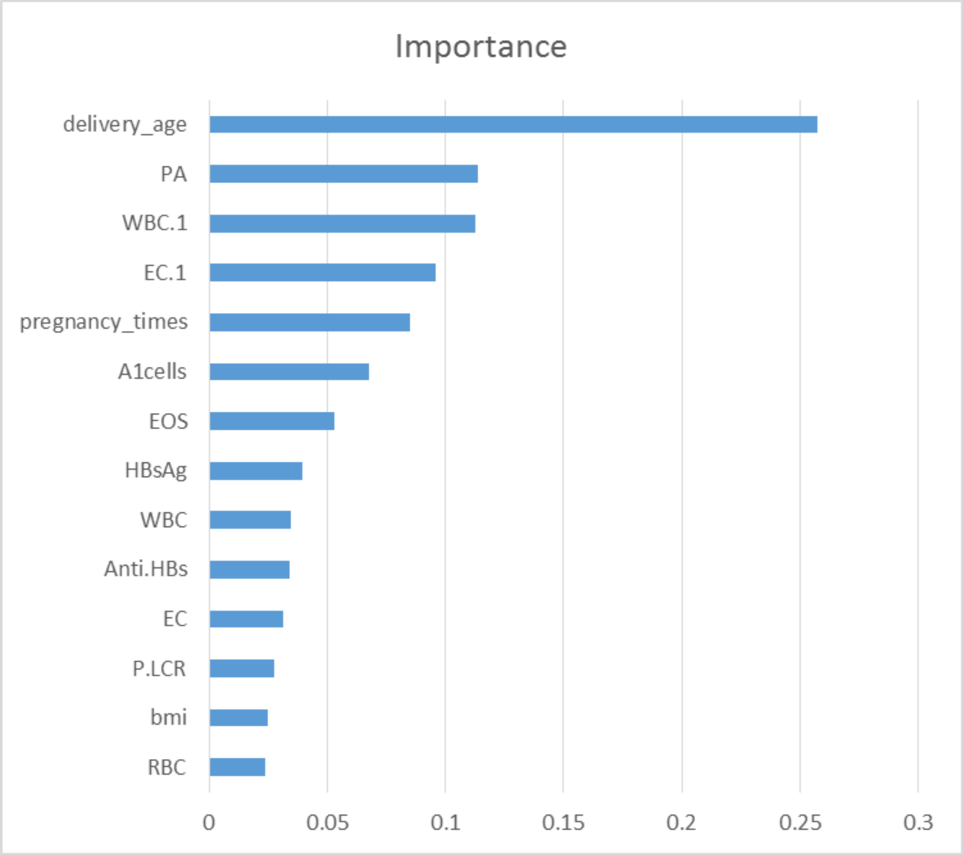

Importance
delivery_age
PA
WBC.1
EC.1
pregnancy_times
A1cells
EOS
HBsAg
WBC
Anti.HBs
EC
P.LCR
bmi
RBC
0
0.05
0.1
0.15
0.2
0.25
0.3

C

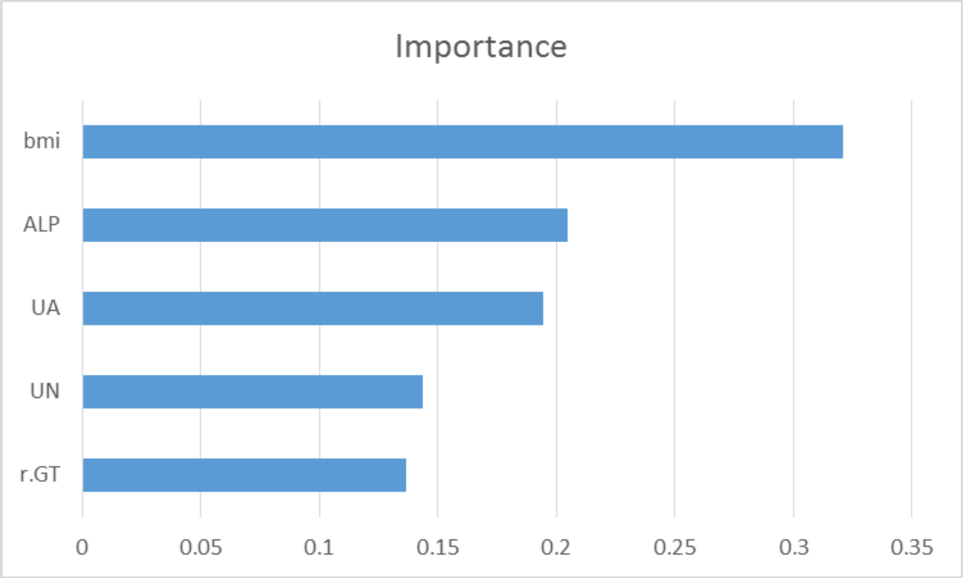

Importance
bmi
ALP
UA
UN
r.GT
0
0.05
0.1
0.15
0.2
0.25
0.3
0.35

D

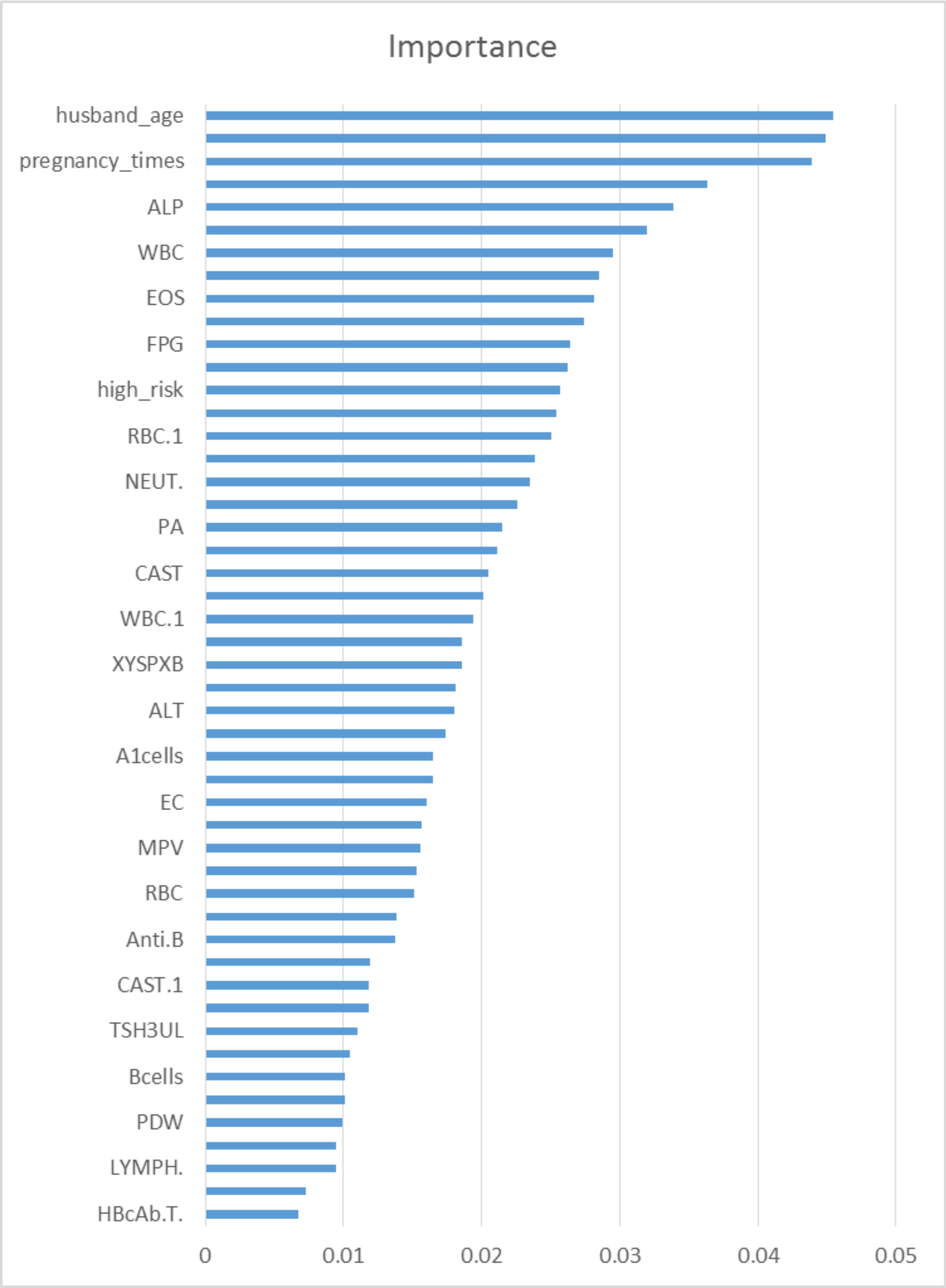
Importance
husband_age
pregnancy_times
ALP
WBC
EOS
FPG
high_risk
RBC.1
NEUT.
PA
CAST
WBC.1
XYSPXB
ALT
A1cells
EC
MPV
RBC
Anti.B
CAST.1
TSH3UL
Bcells
PDW
LYMPH.
HBcAb.T.
0
0.01
0.02
0.03
0.04
0.05

E

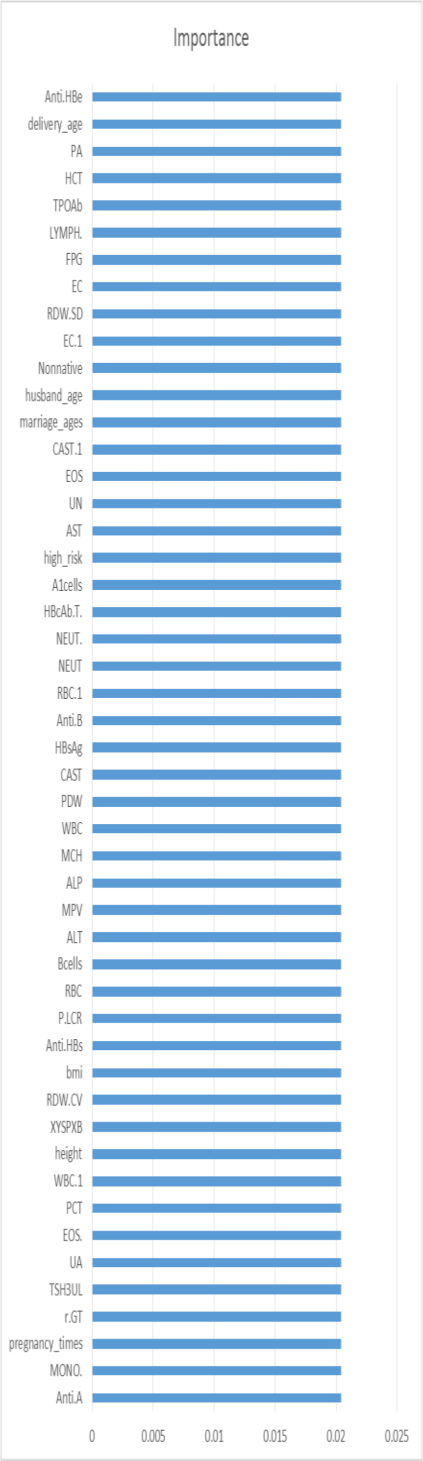

Figure A3. Parameter estimates of input attributes for the prediction models. A:SVM; B CHAID tree, C: Bayesian network, D: Neural network (NN) ; E:CSHM.

Appendix A7.

With the filter method of Pearson's correlation, the values of all the attributes are demonstrated in A4(A). Subsequently, the selected attributes are obtained with the threshold 0.95, as shown figure A4(B). Using those selected attributes, the performance of those methods are achieved, as shown in figure 4(C) and Table A4. When those attributes with their maximum class size greater than 95% (Single category is too large) were removed, the performance of those methods became worse.

A

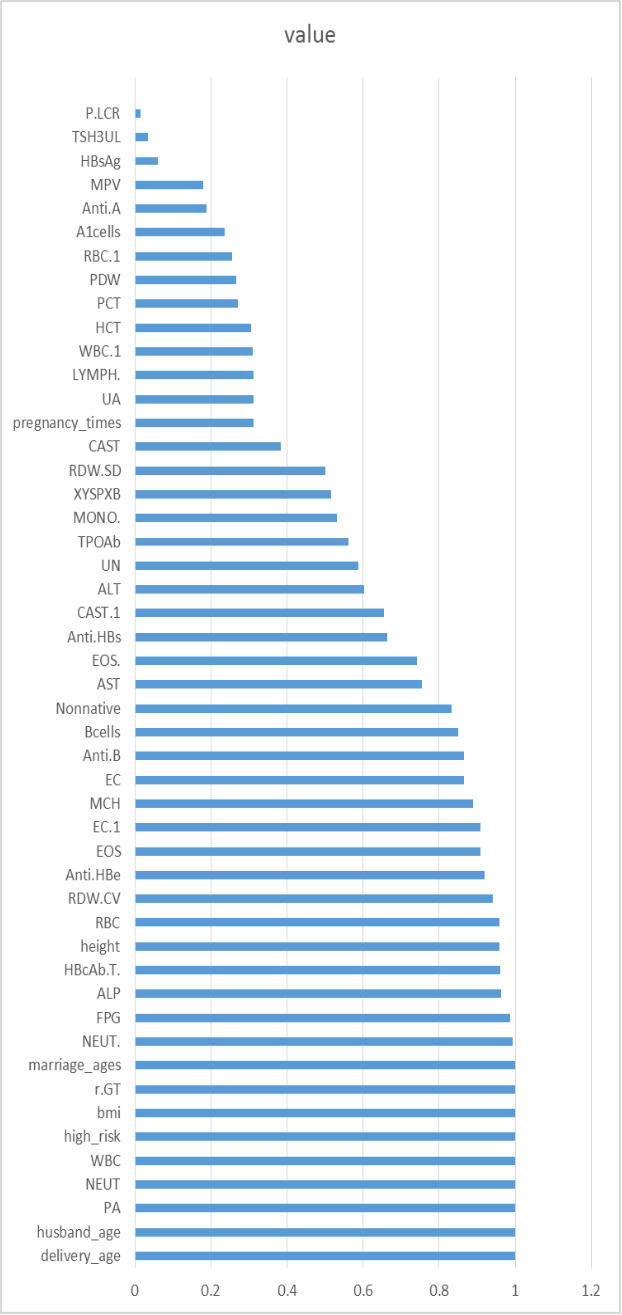

B

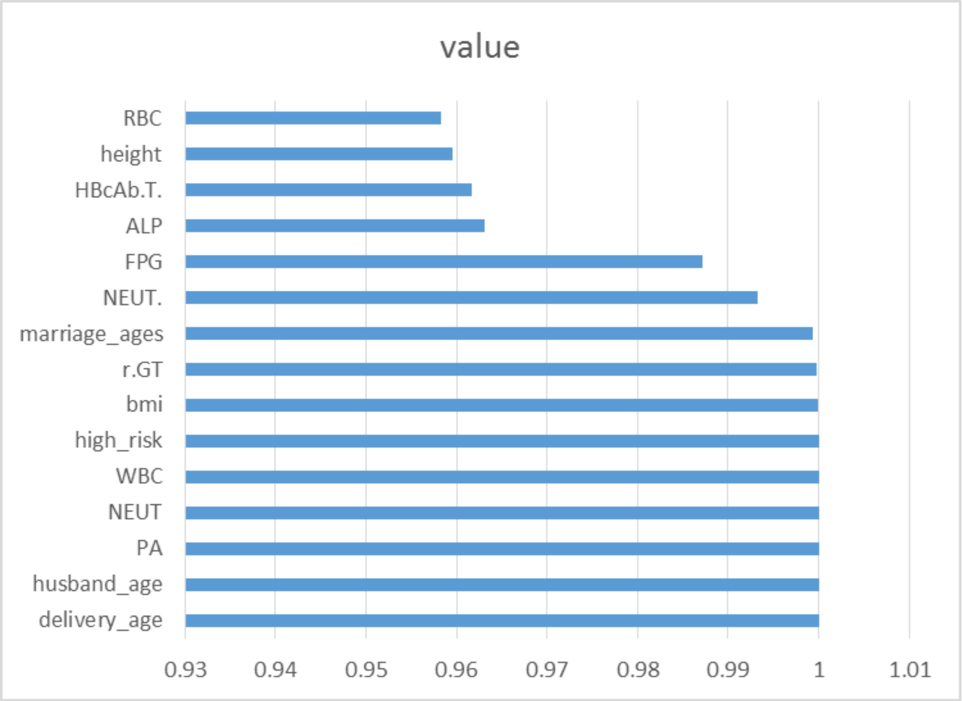

C

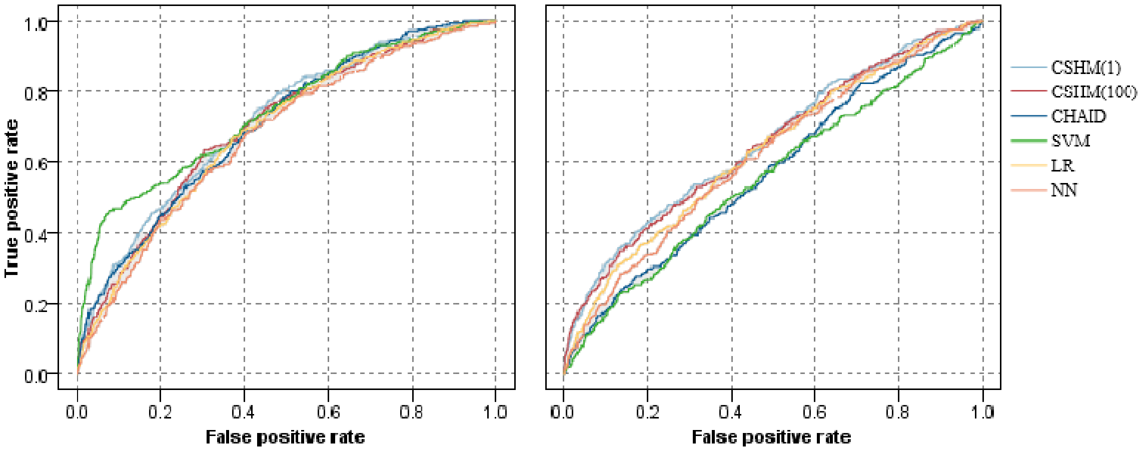

Figure A4. Performance of those methods on selected attributes. A: The Values based on Pearson; B: The selected attributes with their Pearson larger than 0.95; C: Perforamnce of the five methods (except BN) on those selected attributes in trainging (left) and testing (right).

Table 3 Perforamnce of the five methods (except BN) on those selected attributes

Models	AUC	Gini	AUC	Gini
CSHM(1)	0.715	0.431	0.656	0.313
CSHM(100)	0.702	0.404	0.649	0.298
CHAID	0.706	0.411	0.572	0.145
SVM	0.742	0.484	0.558	0.116
LR	0.691	0.382	0.631	0.261
NN	0.676	0.352	0.618	0.235

Reference

1 Zhu, W. W. & Yang, H. X. Diagnosis of gestational diabetes mellitus in China. *Diabetes Care* **36**, e76 (2013).

2 Huttly, W. J., Bestwick, J. P. & Wald, N. J. Insulin Dependent Diabetes Mellitus (IDDM) and first trimester markers in prenatal screening for Down's syndrome. *Prenatal Diagnosis* **36**, 97-103 (2016).

3 Baker, S. G. *et al.* How to interpret a small increase in AUC with an additional risk prediction marker: decision analysis comes through. *Statistics in Medicine* **33**, 3946-3959 (2014).

4 Chen, Q. *et al.* An ontology-driven, case-based clinical decision support model for removable partial denture design. *Scientific Reports* **6**, 27855 (2016).

5 Schapire, R. E. & Singer, Y. Improved boosting algorithms using confidence-rated predictions. *Machine Learning* **37**, 297-336 (1999).

6 IBMKnowledgeCenter. Analysis Output Browser. (2017-9-14). <https://www.ibm.com/support/knowledgecenter/SS3RA7_15.0.0/com.ibm.spss.modeler.help/analysis_browser_analysistab.htm >.

7 Consultation, W. E. Appropriate body-mass index for Asian populations and its implications for policy and intervention strategies. *Lancet* **363**, 157-163 (2004).

8 Alonzo, T. A. Clinical Prediction Models: A Practical Approach to Development, Validation, and Updating By Ewout W. Steyerberg. *Journal of the Royal Statistical Society: Series A (Statistics in Society)* **172**, 661-662 (2009).
